# Nanoimmunotherapy: the smart trooper for cancer therapy

**DOI:** 10.37349/etat.2025.1002308

**Published:** 2025-04-10

**Authors:** Suphiya Parveen, Dhanshree Vikrant Konde, Safal Kumar Paikray, Nigam Sekhar Tripathy, Liza Sahoo, Himansu Bhusan Samal, Fahima Dilnawaz

**Affiliations:** Alma Mater Studiorum Università di Bologna, Italy; ^1^Department of Biotechnology and Genetics, School of Sciences, Jain (Deemed-to-be-University), Bengaluru 560027, Karnataka, India; ^2^School of Biotechnology, Centurion University of Technology and Management, Jatni 752050, Odisha, India; ^3^School of Pharmacy and Life Sciences, Centurion University of Technology and Management, Jatni 752050, Odisha, India

**Keywords:** Nano-immunotherapy, tumor microenvironment, tumor immunity, nanoparticles, nanomaterials, checkpoint inhibitors, CAR-T, CAR-M

## Abstract

Immunotherapy has gathered significant attention and is now a widely used cancer treatment that uses the body’s immune system to fight cancer. Despite initial successes, its broader clinical application is hindered by limitations such as heterogeneity in patient response and challenges associated with the tumor immune microenvironment. Recent advancements in nanotechnology have offered innovative solutions to these barriers, providing significant enhancements to cancer immunotherapy. Nanotechnology-based approaches exhibit multifaceted mechanisms, including effective anti-tumor immune responses during tumorigenesis and overcoming immune suppression mechanisms to improve immune defense capacity. Nanomedicines, including nanoparticle-based vaccines, liposomes, immune modulators, and gene delivery systems, have demonstrated the ability to activate immune responses, modulate tumor microenvironments, and target specific immune cells. Success metrics in preclinical and early clinical studies, such as improved survival rates, enhanced tumor regression, and elevated immune activation indices, highlight the promise of these technologies. Despite these achievements, several challenges remain, including scaling up manufacturing, addressing off-target effects, and navigating regulatory complexities. The review emphasizes the need for interdisciplinary approaches to address these barriers, ensuring broader clinical adoption. It also provides insights into interdisciplinary approaches, advancements, and the transformative potential of nano-immunotherapy and promising results in checkpoint inhibitor delivery, nanoparticle-mediated photothermal therapy, immunomodulation as well as inhibition by nanoparticles and cancer vaccines.

## Introduction

Cancer remains one of the most incurable diseases in the world. The primary treatment options for cancers are radiation, chemotherapy, surgical excision, or a combination of these approaches. Despite these, a great deal of malignancies usually spread before they are discovered, and the majority of surgical resections need the complete removal of the organ, which can be quite damaging to the patient. Further, with high radiation dosages, most tumor cells persist as micrometastases, which are challenging to remove completely. Chemotherapy (e.g., vincristine, camptothecin, anthracyclines, and paclitaxel, etc.) mostly target DNA strands directly using substances like antimetabolites and platinum compounds that prevent DNA replication and cause DNA damage [1, 2]. Chemotherapy unavoidably weakens the patient’s immune system and lowers their quality of life [3]. Immunotherapy has completely changed the way of cancer treatment and has led to a better knowledge of tumors, targeting the entire tumor microenvironment (TME) rather than just the cancer cells [4]. Immune cells have a complex array of mechanisms, tumor cells are degenerated from autologous epithelial cells and, as a result, display very low antigenicity and are not readily recognized by the immune system to detect and eliminate cancer cells [5]. The cancer immune editing, emphasizes the dual role of the immune system in suppressing tumor growth while also shaping tumor immunogenicity and uses a three-step process to describe the process of tumorigenesis (elimination, equilibrium, and escape), which elucidates how some cancer cells, despite being recognizable by the immune system, can evade the attack of the immune system [4]. Immunological evasion is achieved by cancers through a variety of strategies that hijack host-tumor immunological interactions [6]. Immunotherapy, which aims to enhance antitumor immune responses to limit tumor formation, has emerged as an effective cancer treatment strategy [7, 8]. The benefits of immunotherapy in the treatment of cancer are suggested by the clinical success of blocking antibodies that target p programmed cell death-1 (PD-1) and cytotoxic T lymphocyte-associated antigen-4 (CTLA-4) [9, 10]. Over the past two decades, immunotherapy has significantly advanced the treatment of cancer, although it is only beneficial for a tiny percentage of patients [11]. The main thought to be the reason for treatment failure is the development of a tumor immunosuppressive microenvironment (TIME). Thus, enhancing TIME is a sophisticated tactic to enable the clinical use of cancer immunotherapy, which can stimulate antitumor immune responses to eliminate tumors [12, 13].

While some immunotherapies specifically target certain tumor antigens, others generally stimulate the immune system. Although many types of immunotherapeutic drugs have become available, intrinsic limitations associated with drug delivery, dose-limiting toxicity, poor tumor permeability, low uptake rates, and low response rates have hindered the widespread application of immunotherapeutic drugs. Immunotherapy side effects might range from minor and localized to more severe and systemic because of this heterogeneity [14, 15]. Some of the challenging issues like, the development of resistance to cancer immunotherapies, the inability to predict treatment efficacy, patient response, the need for additional biomarkers, the absence of clinical study designs that are optimized to determine efficacy, along with high cost of treatment [16, 17]. Further, despite significant advancement, the clinical application of immunotherapies still faces several obstacles to its safety and efficacy.

Nanoparticles (NPs) based cancer immunotherapy using biomaterials could efficiently used for the creation of several kinds of NPs for combinational immunotherapies, in terms of reprogramming TMEs and boosting antitumor immune responses, increasing their potency, and lessening harmful side effects [18]. NPs are creating new opportunities for combining cancer immunotherapy with traditional treatment modalities to amplify the antitumor immune responses [19]. In this review, recent advancements in NP-mediated approaches, the challenges and possibilities of integrating delivery systems into cancer immunotherapy, and their prospects are discussed.

## Impediments of tumor immunity and physiological barriers to nanomedicine access

Cancer immunotherapy has perceived remarkable gains due to recent developments in fundamental immunology, which has motivated oncologists to apply this knowledge for the treatment of cancer therapy. Nevertheless, several obstacles restrict immunotherapy’s ability and affect patients’ survival. Tumor cells have several defense mechanisms against the immune system’s reaction. Evasion of the immune system is caused by a combination of the expression of inhibitory markers and the transformation of cellular infiltrates that enable the cell to tolerate. Furthermore, certain tumor cells trigger the immune system to autoreact to host tissue, while others develop resistance to apoptosis through other means. The efficacy of immunotherapy and tumor regression are both impeded by these pathways [20].

The physicochemical features of nanomedicines (composition, size, shape, charge, surface modification, etc.) and the methods of administration considerably affect their pharmacokinetics (PKs) [21]. These nanoscale medications have distinct PKs following systemic delivery in contrast to small molecular medicine. They have a longer blood circulation time and are more likely to evade excretion through the kidney and be captured by the reticuloendothelial system (RES) in the spleen, liver, and lung, which leads to increased tumor buildup. However, because of the body’s numerous physiological obstacles, conventional nanomedicines continue to face issues with poor delivery efficacy and unsatisfactory therapeutic effects [22]. In addition to the physiological obstacles for nano delivery methods, a significant obstacle for cancer immunotherapy that leads to poor response rates is the immunosuppressive microenvironment [23]. The anticancer response mediated by immune checkpoint inhibitors (ICIs) depends on T lymphocyte infiltration into the body. In cold tumors, the immune system cannot effectively target and destroy because, there is little to no immune infiltration surrounding the cancer cells, making ICIs ineffective against them. Therefore, it is necessary to find out the mechanism of the tumor environment of cold tumors [24]. On the other hand, in hot tumors, a lot of immune cells have infiltrated surrounding the cancer cells, and the cancer cells themselves emit chemicals that draw in immune cells and trigger the immune response. Usually, immunotherapy is effective against this kind of cancer. One of the challenges faced during the treatment of cold tumors is usually the scarcity of efficient antigens that serve as targets for immunotherapy. Cold tumor patient’s cancer cells have few or no antigens on their surface, immune cells find it challenging to recognize and to successfully combat the malignancy [25]. ICIs may have a better therapeutic impact if cool tumors are tuned into hot tumors [26]. Many strategies have been proposed to boost T cell trafficking, infiltration, and T cell expansion by reorganizing the tumor immune microenvironment and encouraging T cell priming and activation by increasing antigen processing and presentation [27]. NPs can reach specific immune cells or receptors by surface functionalization with ligands and peptides and coating with polyethylene glycol (PEG). They can also delay immune system detection and elimination of the NPs, extending their time in the circulatory system [28, 29]. By altering the behavior of cytokines and signaling molecules that promote immune cell communication, NPs can have an impact on immunological responses. Depending on their unique characteristics, NPs can either stimulate or hinder the synthesis of cytokines [30]. Numerous materials have been investigated as the basis for NP production in nucleic acid delivery. Polymeric and dendrimeric materials, natural and synthetic lipid-based substances, peptide/protein-derived biomolecules, inorganic frameworks [31], and, more recently, exosomes [32]. Neutral biomaterials have been utilized for straightforward nucleic acid entrapment or encapsulation, whereas cationic biomaterials use the anionic nature of the nucleic acids to create ionic complexes for NP synthesis and/or trapping. Certain NPs are made from a single (homogenous) component, which makes synthesis easier but may restrict the NPs’ functional characteristics. Regardless of the building block, the NPs can help deliver nucleic acids into cells by a variety of uptake mechanisms, such as receptor-mediated internalization and cell membrane penetration, and by further binding to nucleic acids to shield them from extracellular environment degradation [33].

**Figure 1:**
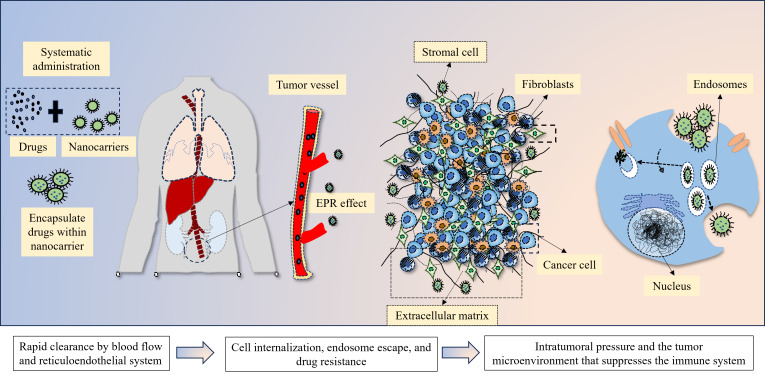
The biological barriers for nanocarriers for delivering drugs to tumors, through enhanced permeability and retention (EPR) effect

Nanomedicines must pass through several successive barriers when administered systemically before they can effectively reach the tumor locations (Figure 1) [34, 35]. The first barriers to nanomedicines following injection are the RES’s quick uptake and clearance of blood flow, which typically results in a loss of over 99% of administered [36, 37]. The foremost hindrances that nanomedicines face during systemic circulation are renal excretion, RES mononuclear phagocyte system absorption, and enzyme breakdown. Importantly, because serum proteins cover them, nanomedicines in the circulation have trouble forming protein coronas. This causes non-specific accumulation and adverse effects by deactivating the ligand’s targeting capacity and facilitating macrophage absorption in the mononuclear phagocyte system impacting vital organs such as the lung, spleen, and liver [38, 39]. Additionally, the blood flow affects the stability of nanocarriers and typically results in payload burst release. The suspension of nanocarriers from tumoral vessels to tumor tissues and the deep penetration of nanocarriers within tumors are hindered by high intratumoral pressure, which is linked to disrupted blood vessels, aggressive tumor cell proliferation, stroma cells, tumor-associated fibroblasts, and the extracellular matrix (ECM) [40, 41]. Endosome escape and cellular internalization are also crucial obstacles that prevent nanomedicines from reaching their therapeutic effects once they reach the tumor cells. Inaptly, the “PEG dilemma”, which refers to the issue of poor uptake by the targeted cells, affects the majority of nanomedicines with long blood circulation qualities [42]. Further, the development of protein coronas increases the possibility of off-target consequences for nanomedicines containing active-targeting ligands [43]. Furthermore, drug resistance brought on by drug efflux pumps has shown to be a significant barrier for nanomedicines [44, 45]. The clinical transition of nanomedicines from bench to bedside is significantly hampered by these biological hurdles. The enhanced permeability and retention (EPR) effect was proposed by Maeda in the 1980s, which explained the uptake of nanomedicines [46]. Passive targeting of nanocarriers relies on the EPR effect-based accumulation in malignancies [46]. Further, these nanocarriers can be made stealthily with a coating of PEGs and zwitterionic polymers [47]. Current marketed NP-based anticancer drugs all rely on passive targeting pathways to accumulate in tumors. However, a meta-analysis of 2,589 patients in the clinic revealed that the liposomal DOX did not increase objective response, overall survival, or progression-free survival rates [48]. Unspecific delivery and the extremely variable EPR impact in individuals may be the cause of nanomedicines’ meager clinical results [46, 49]. Responses to the EPR effect vary among patients, cancer kinds, and even within a single patient, distinct tumoral lesions. Ligand-functionalized nanocarriers for active tumor targeting have been created as the second generation of nano-scale drug delivery systems to increase anticancer efficacy. Both the EPR effect and strong bind affinity to the particular biomarkers on the targeted cancer cells and tumor vascular epithelial cells are necessary for these active targeting nanocarriers to reach the tumor locations [50, 51]. Different small molecules and biomolecules are used as targeting ligands [52]. There are at least 15 ligand-conjugated nanocarrier-based nanomedicine formulations are in clinical trials, including nine liposomal formulations (MM-302, C225-ILSDOX, anti-EGFR-IL-dox, SGT-53, SGT-94, Lipovaxin-MM, MCC-465, 2B3-101, and MBP-426), two bacterial-derived minicells [TargomiRs and EGFR(V)-EDV-Dox], two polymeric NPs (BIND-014 and CALAA-01), one retroviral vector (Rexin-G), and one NP-based vaccine for smoking cessation (SEL-068) [53]. However, the antibody-drug conjugates (ADCs) display remarkable clinical success [54, 55]. Nanomedicine has shown many advantages in animals, but most clinical studies have shown that nanomedicine does not show therapeutic advantages, such as prolonging survival time and improving cure rate, but only changes in drug absorption, distribution, metabolism, and toxicity. Establishing appropriate and trustworthy models that are closer to human tumoral settings and creating non-invasive companion nanodiagnostic devices to track the therapeutic results are crucial for the effective clinical translation of actively targeted nanomedicines.

NPs with endosomal escape mechanisms are preferred when constructing the carriers because endosomal membranes pose a significant obstacle to the movement and release of nucleic acids. This has been accomplished through a variety of methods; like endosomal entrapment could be prevented by employing “fusogenic” NPs, which can fuse with the cell membrane and release their payload into the cytoplasm [56]. The three peptide variations that were created, DIVA3, DIV3H, and DIV3W, were able to bind to short interfering RNA (siRNA) in monodisperse NP complexes and shield siRNAs from RNase and serum destruction. To lessen the aggressiveness of ovarian cancer siRNA targeting casein kinase II (CSNK2A1) is delivered via fusogenic peptide carriers. Compared to non-targeting siRNAs, peptide DIV3W induced up to 94% suppression of CSNK2A1 mRNA and showed effective transport of bioactive siRNAs into ovarian cancer cells with high cellular uptake efficiency, which reduced cell migration and recolonization in vitro. In subcutaneous ovarian tumors, intramoral administration of DIV3W-siCSNK2A1 complexes led to decreased CK2α protein expression and CSNK2A1 mRNA after 48 h, as well as decreased tumor growth and migration after a 2-week multi-dosing regimen [57]. NPs have a variety of ways to influence the immune system. A key mechanism is the transfer of antigen to antigen-presenting cells (APCs), such as dendritic cells (DCs). To increase T cell activation and the consequent adaptive immune response, antigens can be encapsulated in NPs and delivered straight to APCs. Since NPs can carry antigens and deliver them to the immune system accurately and efficiently, this approach is highly beneficial when creating vaccines [58]. NPs may play an adjuvant role and affect the immune system. Adjuvants are compounds that enhance the immune system’s response to an antigen. Through the creation of a retention effect and the subsequent progressive release of the antigen, NPs can function as adjuvants, extending the immunological response. It is also possible to add pathogen-associated molecular patterns (PAMPs), which are recognized by immune cell pattern recognition receptors (PRRs), to their surface. This identification triggers innate immune responses, which in turn enhance the antigen-specific immune response [59]. Additionally, NPs can be used to precisely deliver drugs or immunomodulatory chemicals to the targeted immune cells. Immune cell activity can be controlled with this tailored delivery to either enhance or decrease function as needed [60]. However, the body’s fight against cancer can be strengthened by the immunostimulatory chemicals present in NPs, which can boost immune cell activity [61]. The blood-brain barrier, a highly selective membrane that shields the brain from dangerous substances, is one biological barrier that NPs can also pass through [62].

## Tumor immunity and efferocytosis in the TME

TME consists of diverse immune-associated cells like tumor-associated fibroblasts, endothelial cells (ECs), pericytes, and other tissue-resident cells. These host cells are important players in the pathophysiology of cancer, and once were thought to be bystanders of carcinogenesis [63]. The TME depends on the organ where it develops, the intrinsic properties of cancer cells, the stage of the tumor, the cellular makeup, and its functional status [64]. Although the TME’s makeup varies depending on the kind of tumor, immune cells, stromal cells, blood vessels, and ECM are all common components. “TME is not just a silent bystander, but rather an active promoter of cancer progression”, according to popular belief [65]. There are two types of immune cells: innate immune cells and adaptive immune cells. Exposure to particular antigens triggers adaptive immunity, which then employs an immunological memory to “evaluate” the threat and strengthen immune responses. The adaptive immune response is made up of T-cells, B-cells, and natural killer (NK) cells. Within hours of a foreign antigen entering the body, innate immunity—a general defense mechanism—kicks in. DCs, neutrophils, and macrophages are among the cells that execute an innate immune response [65]. Cytotoxic T-cells (CD8^+^) identify aberrant tumor antigens on cancer cells and destroy the tumor cells. In cancer patients, the presence of cytotoxic T-cells in the TME is frequently linked to a favorable prognosis. By secreting interferon-gamma (IFN-γ), cytotoxic T-cells not only destroy tumor cells but also inhibit angiogenesis. Within the framework of the TME, CD4^+^ T-cells coordinate a broad spectrum of immunological responses by differentiating into distinct subtypes. T helper 1 (Th-1) cells are proinflammatory CD4^+^ T-cells that secrete IFN-γ and interleukin-2 (IL-2) to assist CD8^+^ cells. Several cancer types are linked to elevated Th-1 cell counts inside the TME. Regulatory T cells (Tregs) are needed to regulate autoimmunity and inhibit inflammatory reactions. Tregs are common in the TME, they suppress the antitumor immune responses and support the growth and spread of tumors [66, 67]. Tregs have a dual role because they suppress immune responses in many illness contexts (pathological role) and maintain immunological homeostasis (protective role). They decrease the actions of T effector cells (Teff), which aids in the initiation and spread of cancer. A poor prognosis for the majority of cancer types has been linked to decreased intratumoral CD8^+^ T cell-to-Treg ratios. Using ICIs to target immunological checkpoints (ICs), such as CTLA-4 and PD-1, has been shown to improve clinical outcomes and induce anti-tumor immune responses in cancer patients [68]. In immune-excluded tumors, immune cells, particularly cytotoxic T lymphocytes (CTLs), are restricted to the tumor periphery and fail to infiltrate the tumor core. This exclusion is often mediated by physical barriers such as dense ECM components and the presence of cancer-associated fibroblasts (CAFs), which impede immune cell penetration. Consequently, patients with immune-excluded tumors typically exhibit a poorer prognosis and diminished responses to immunotherapies, including ICIs, due to the inability of effector immune cells to reach and eradicate tumor cells. Conversely, immune-invaded tumors are characterized by substantial infiltration of immune cells, especially CTLs, within the tumor parenchyma. This infiltration correlates with a more favorable prognosis and enhanced responsiveness to immunotherapy, as the presence of effector immune cells within the tumor facilitates effective anti-tumor immune responses [69].

Moreover, cytosolic DNA-mediated STING pathway activation enhances antigen presentation and T-cell priming for anti-tumor immunity by stimulating the synthesis of type I interferons and pro-inflammatory cytokines. Effective tumor cell killing is made possible by checkpoint inhibition, which targets molecules such as CTLA-4 and PD-1/programmed death-ligand 1 (PD-L1) to restore depleted T-cell function. Toll-like receptor (TLR) signaling primes adaptive immunity and activates DCs, especially through TLR7/8 and TLR9. Cytokines including IL-2, IL-12, IL-15, and IFN-γ promote the growth and activation of NK cells and CTLs, which in turn support immunological activation. Agents such as anti-CD25 antibodies can be used to modify Tregs to counteract immunological suppression. STAT3 targeting can be used to inhibit myeloid-derived suppressor cells (MDSCs), which affect the function of T-cells and NK cells [70].

Specialized immune cells called B-cells are in charge of producing antibodies, presenting antigens, and secreting cytokines. B-cells are frequently observed in lymph nodes near the TME and tend to concentrate toward the tumor’s edge. There are comparatively fewer infiltrating B-cells in the TME than T-cells. Infiltrating tumor “tertiary lymphoid structures” are ectopic lymphoid structures that arise within the TME, and B cells play a key role in their production. Tertiary lymphoid structures are a good indicator of prognosis and enable tight T-B cell interaction. B-cells’ anti-tumorigenic functions include presenting antigens to T-cells, producing anti-tumor antibodies, and secreting cytokines that stimulate cytotoxic immune responses (such as IFN-γ). On the other hand, B-cells may have protumor effects, and their presence in the TME may indicate a bad prognosis for renal cell carcinoma, bladder cancer, and prostate cancer. By producing cytokines including transforming growth factor-beta (TGF-β) and IL-10, which encourage immune-suppressive traits in neutrophils, macrophages, and cytotoxic T cells, regulatory B-cells encourage tumor aggression [67, 71]. NK cells search for tumor cells or host cells infected by viruses in the bloodstream. Two functional classes are involved, out of them one releases inflammatory cytokines and the other takes part in cell-mediated destruction of tumor cells. NK cells prevent metastasis by eliminating tumors in the bloodstream, however, they are less effective in eliminating tumor cells in the TME [72]. Macrophages can strongly infiltrate in some tumor types, accounting for as much as 50% of the tumor’s bulk. In the TME, macrophages frequently encircle blood vessels, secreting vascular endothelial growth factor (VEGF)-A, promoting the creation of new blood vessels [73]. Macrophages play a pivotal role in tumor development and progression, exhibiting dual functions depending on their polarization and interaction with the TME. Tumor-associated macrophages (TAMs), often polarized toward the M2 phenotype, promote tumor growth by secreting anti-inflammatory cytokines (e.g., IL-10, TGF-β) and pro-angiogenic factors (e.g., VEGF), facilitating immune evasion, angiogenesis, and metastasis [74]. They also suppress cytotoxic T-cell activity, recruit Tregs, and release matrix metalloproteinases (MMPs) that remodel the ECM to enable tumor invasion. Conversely, M1 macrophages exhibit anti-tumor properties by producing pro-inflammatory cytokines [e.g., IL-12, tumor necrosis factor-alpha (TNF-α)], releasing cytotoxic agents like reactive oxygen species (ROS) and nitric oxide (NO), and presenting tumor antigens to activate T cells. Clinically, targeting TAMs to reprogram them from M2 to M1 phenotypes or inhibit their recruitment holds promise for reducing tumor progression. Additionally, TAM polarization status is being explored as a biomarker and therapeutic target for cancer treatment [75].

Neutrophils are the initial line of defense against many infections, these neutrophils can either prevent or encourage tumor growth. During the growth of the tumor, neutrophils are drawn to the TME and cause inflammation by releasing ROS and cytokines that encourage tumor cell death. In later stages of tumor development, neutrophils increase angiogenesis, which in turn leads to tumor progression and local invasion by altering the ECM, releasing VEGF, and manufacturing MMP9 [76]. DCs are the APCs essential to the immune system because they identify, seize, and deliver antigens to T-cells. DCs initiate pathogen-specific T-cell responses by bridging the gap between innate and adaptive immunity by supplying environmental cues that either accept or trigger an immune response to tumor cells, the TME. Cancer cells recruit supporting cells from nearby endogenous tissue stroma to promote critical steps in tumor formation. Vascular ECs, fibroblasts, adipocytes, and stellate cells are among the stromal cell types, which can differ greatly throughout tumor types. After being drawn to the TME, stromal cells release a variety of substances that affect angiogenesis, invasion, proliferation, and metastasis [77].

Vascular endothelium (VE), a thin layer of ECs, aids in the coordination of blood vessel development. VE not only keeps circulating blood away from tissues, but it also transports immune cells, supplies water and nutrition, keeps metabolic homeostasis stable, and helps create new blood vessels. Cancer cells use passive diffusion to exchange gases and move nutrients during growth. The tumor develops their own blood supply, by activating hypoxia-inducible factors (HIFs), transcription factors critical in coordinating cellular responses to low O_2_. VEGF promotes EC migration to generate new blood vessel lumens in both autocrine and paracrine ways. After that, ECs release proteins to create fresh basement membranes. Early stages of tumor growth are characterized by leaky vasculature, which is caused by blood vessels in the TME frequently failing to reach the final stages of maturity [78]. Further, in addition to angiogenesis ECs play a crucial role in encouraging cancer cell motility, invasion, and metastasis, due to their great degree of plasticity. ECs change into CAFs through an endothelial-mesenchymal transition as tumors grow. Bone morphogenetic protein (BMP) and TGF-β coordinate the change from an EC to a CAF, which results in increased migration, detachment and elongation, loss of endothelial characteristics, and a loss of cell-to-cell contacts [28].

Metastasis is a multi-step process, in which there is translocation of cancer cells from the main TME to distant areas through intravasation. CAFs are a significant part of the tumor stroma and are essential for promoting communication between TME and cancer cells. CAFs have a variety of origins, however, tissue-resident fibroblasts are frequently the source. Additionally, adipocytes, ECs, pericytes, stellate cells, and bone marrow-derived mesenchymal stem cells create CAFs. Myofibroblasts, actively aid in wound healing and can be reversibly produced from fibroblasts that ordinarily exist within tissues upon injury. TGF-β signaling activates myofibroblasts, which then go on to acquire traits like proliferation, contractile qualities, secretory phenotypes, and ECM synthesis that are crucial for wound healing. Tumors have been aptly termed “wounds that never heal” [29]. Adipocytes also play an important role in modifying ECM through secretion of metalloproteases. It secretes metabolites, enzymes, hormones, growth factors, and cytokines, adipocytes that influence the TME. Adipocytes and tumor cells interact dynamically and reciprocally inside the TME to promote the growth of tumors. Stellate cells are mesenchymal stromal cells that are quiescent and found in the pancreas and liver. During their quiescent state, pancreatic stellate cells (PSCs) produce degradation enzymes and ECM proteins including desmin and vimentin, which aid in the alteration of ECM. PSCs are activated by vitamin A deficiency, which increases their capacity for migration and proliferation and causes them to secrete cytokines and chemokines [79]. Death cell removal is essential for illness, tissue repair, and homeostasis [80]. Efferocytosis, the process by which phagocytes consume dead cells, is mostly carried out by macrophages [81]. Macrophages are essential immune cells that can adapt to different situations, taking either supporting or tumor-repressive roles [82]. Many inflammatory diseases, such as infections, cancer, and atherosclerosis, are linked to abnormalities in efferocytosis [83].

When an infection or tissue damage occurs, neutrophils are the initial cellular innate immune response that rapidly gathers at the site of tissue injury through the multi-step mechanism of “neutrophil swarming” [84]. Fridlender et al. [85] distinguished between two groups of tumor-associated neutrophils (TANs): anti-tumourigenic N1-TANs and protumorigenic N2-TANs. In numerous human malignancies, protumourigenic N2-TANs are present [86]. It secretes a range of growth hormones, cytokines, and chemokines that support the survival and multiplication of tumor cells, including VEGF, prostaglandin E2 (PGE2), CCL17, IL-6, TNF-α, and epidermal growth factor (EGF). Further, collagenase (MMP8) and gelatinase B (MMP9), which facilitate the invasion of tumor cells are also secreted by N2-TAN [87]. Neutrophils are essential for efferocytosis when there are large collections of the apoptotic dead cell remains. Neutrophils promote the growth and dissemination of tumors in both the bloodstream and the tissue [88]. Clusters of circulating tumor cells (CTCs)-neutrophils promote tumor cell survival, proliferation, and cell cycle progression, which increases the likelihood of metastasis [89]. Clusters of CTC-neutrophils may also contain neutrophil extracellular traps (NETs), which encourage CTC attachment and extravasation at the metastatic site [90]. For CTCs, the bloodstream is a hostile environment, and individual tumor cells may perish rapidly. Neutrophils have several receptors for the identification and binding of dead cells, and they have the entire apparatus for engulfment [90].

**Figure 2:**
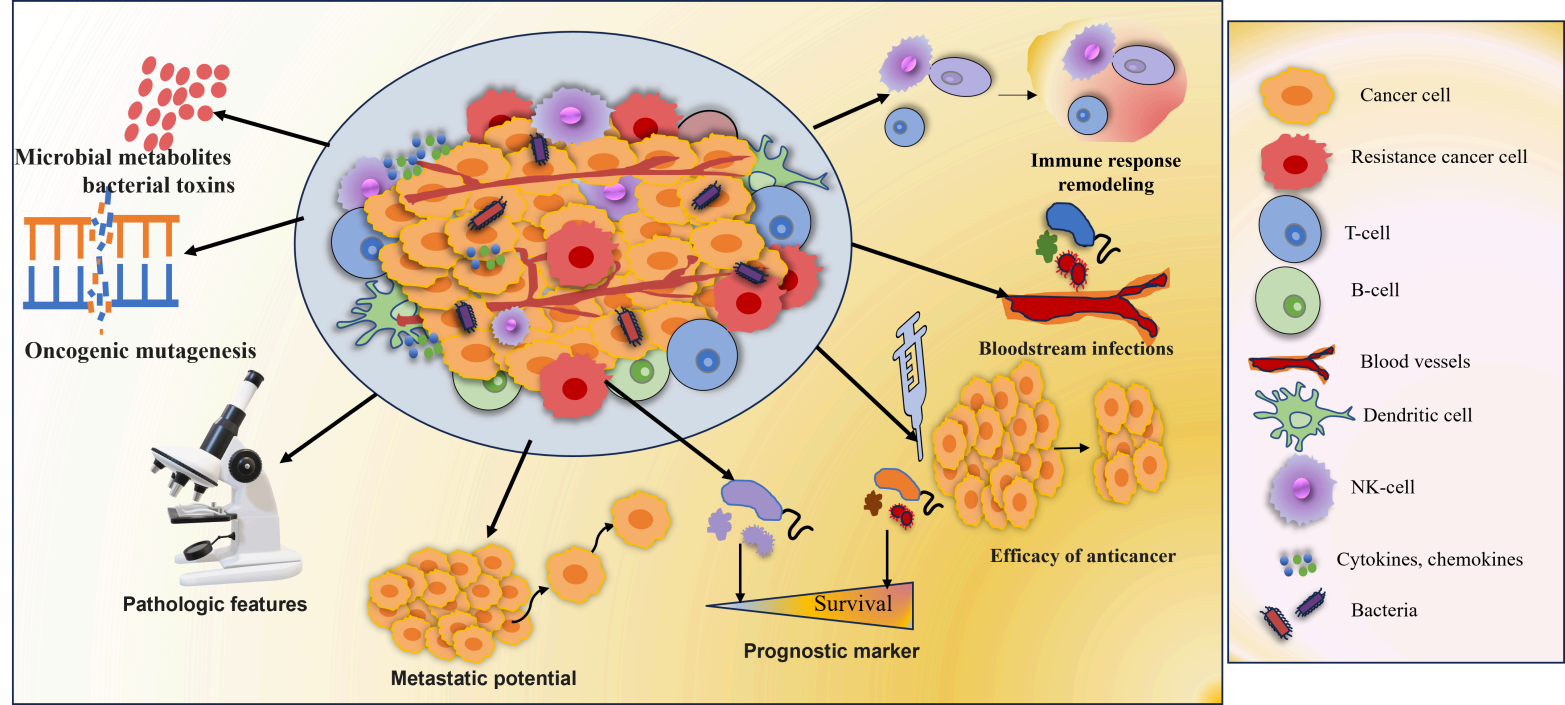
**Microbial metabolites influencing tumor characteristics**. Tumor microbiota plays a significant role in the onset and management of cancer. Increased mutagenesis, control of oncogenes and oncogenic pathways, alteration of host immune response pathways, metabolism of cancer drugs, and the generation of bacterial toxins and microbiota-derived metabolites influence tumorigenesis, cancer progression, and response to therapeutic agents. Growing data from clinical research and animal models demonstrated the link between the tumor microbiome and clinicopathologic characteristics [91] *Note.* Adapted from “Tumor microbiome - an integral part of the tumor microenvironment” by Ciernikova S, Sevcikova A, Stevurkova V, Mego M. Front Oncol. 2022;12:1063100 (https://www.frontiersin.org/journals/oncology/articles/10.3389/fonc.2022.1063100/full). CC BY.

With the development of high-throughput sequencing technologies, there is a shred of increasing evidence that tumor tissue has a microbial ecology (Figure 2). There are several ways through which bacteria can get to tumor cells, by invading mucosal membranes, the circulation, or the gut-organ axis. The bacteria influence the host’s immune system, stimulate inflammation, control metabolism, and initiate invasion and transfer, among other processes, to promote the onset and spread of cancer [91]. The host gut microbiota has a major influence on the TME’s shape, via altering mechanisms involved in tumor promotion. In addition, microbiota-derived metabolites can enter the TME via circulation and become a part of the microenvironment [92]. Wnt/β-catenin signaling is one of the carcinogenic signaling pathways that are activated by tumor-associated microbiota, which aids in carcinogenesis. Various studies indicated that toxin Bft, adhesin A (FadA), cytotoxin-associated gene A (CagA) protein is secreted by *Helicobacter pylori*, *Fusobacterium nucleatum*, and enterotoxigenic *Bacteroides fragilis*, promotes carcinogenesis as well as the innate and adaptive immune responses that are inhibited by intratumoral microbiota [93]. Nejman et al. [94] verified the presence of bacteria in seven solid tumor types like, glioblastoma multiforme (GBM), breast, ovary, bone, pancreas, melanoma, and lung cancer. The study revealed bacterial lipopolysaccharide (LPS) and 16S rRNA in all tumor types. The microbiome of breast tumors was more diverse than the microbiome of other tumor types. While the Actinobacteria phylum, which includes the Corynebacteriaceae and Micrococcaceae families, predominated in non-gastrointestinal cancers, Proteobacteria and Firmicutes were most prevalent in all tumor types [94]. It was also observed that tumor samples from non-responders had higher *Gardnerella vaginalis* abundances [94]. In colorectal cancer patients, the modulation of the innate immune system by *Fusobacterium nucleatum*’s, plays a significant role in chemoresistance. Preclinical research found a link between *Fusobacterium*-mediated resistance to chemotherapeutics (oxaliplatin and 5-fluorouracil) and autophagy modification via the TLR4 and MYD88 signaling pathway [95]. The intratumoral Enterobacteriaceae and Pseudomonadaceae families were found to be in high abundance in pancreatic cancer tissues from pancreatic ductal adenocarcinoma (PDAC) samples [96]. Gammaproteobacteria were able to enzymatically inactivate gemcitabine by expressing the bacterial cytidine deaminase. The antibiotic treatment with ciprofloxacin overcame the gemcitabine resistance [97]. Gemcitabine mycoplasma-infected tumor cell lines demonstrated less cytostatic effect when *Mycoplasma hyorhinis* was present in the TME [98]. The categorization of cancer patients based on the characterization of tumor microbiome characteristics may lead to the development of more specialized, tumor-specific treatments. The advancement of machine learning algorithms might be applied for the identification of underlying mechanisms and signaling networks to identify novel targets for predicting therapy response [91].

## Vaccines for cancer

Conventional cancer vaccines, despite their innovative nature, have frequently encountered difficulties with specificity, potency, and the capacity to produce strong and long-lasting protection. Cancer nanovaccines, on the other hand, use the accuracy of nanotechnology to improve antigen presentation, increase delivery, and alter the TME. Several factors frequently undermine the efficiency of cancer vaccines, most notably tumor-induced immunosuppression and insufficient immune activation brought on by inefficient APC engagement [99]. A tumor-specific antigen is a protein that is exclusive to cancer cells and absent from healthy ones. Antigens unique to tumors can aid the body’s immune response against cancerous cells. They may be employed as potential targets for immunotherapy, which helps to strengthen the immune system and destroy more cancer cells, or as targets for targeted treatment [100]. Numerous cancer immunotherapeutic approaches have been developed using DCs, originating from the initial studies on the generation of ex vivo DCs from mice, beginning with bone marrow precursors. This approach was later extended to humans, utilizing CD34^+^ hematopoietic progenitors or monocytes derived from peripheral blood [101]. Prostate cancer is the focus of the Sipuleucel-T (ProvengeTM) cancer vaccine, which is based on “immune cells” and uses an autologous entire immune cell population treated with PA2024 (a prostate antigen that contains prostatic acid phosphatase, or PAP) linked to GM CSF (granulocyte-macrophage colony-stimulating factor). The United States Food and Drug Administration (US FDA) authorized the first therapeutic cancer vaccine in 2010 intended to treat asymptomatic metastatic castrate-resistant prostate cancer (mCRPC). However, there was no difference in the time to progression, and the median survival of the active treatment group improved by only 4.1 months as compared to the placebo arm [102]. The poxviridae family contains the first and most thoroughly studied viral-based vectors in cancer vaccine trials, including vaccinia, modified vaccinia strain Ankara (MVA), and avipoxviruses (canarypox and fowlpox; ALVAC). It is well known that platelets interact with CTCs and build up at surgical sites. These features make them appealing delivery systems for the targeted administration of immunotherapies and chemotherapy to tumors. The delivery of PD-L1 blocking antibodies to operating rooms and CTCs has been investigated using platelets. ICIs have transformed the treatment of cancer by enabling the immune system to identify and eliminate malignant cells. Immune checkpoint proteins that typically suppress immune responses, such as PD-1, PD-L1, and CTLA-4, are the main targets of this strategy [103]. ICIs suppress T-cell activation by obstructing inhibitory signals. Nevertheless, several variables, including the TME, the existence of immune-suppressive cells, and the drug’s PKs, may restrict its effectiveness. Several cutting-edge delivery techniques have been developed to increase the effectiveness and lessen the negative effects of ICIs [104].

Nanomedicines present special chances to boost these vaccinations’ effectiveness. To increase the strength and longevity of anti-tumor immunity while lowering unfavourable side effects, a range of nanoplatforms have been studied to deliver molecular, cellular, or subcellular vaccines to target lymphoid tissues and cells [105]. With the benefits of a nano-sized range, high antigen loading, improved immunogenicity, regulated antigen presentation, increased retention in lymph nodes, and patient compliance through reduced dose frequency, “nanovaccines” have been investigated to elicit a robust immune response. Different kinds of NPs with different pathogenic or foreign antigens can aid in overcoming immunotolerance and reducing the need for booster shots, which are necessary for traditional vaccinations. Long-lasting immunogenic memory can be produced by nanovaccines, which can also elicit cell-mediated and antibody-mediated immunity [106, 107].

NPs, such as liposomes and polymeric carriers, ensure tumor-specific delivery of ICIs like anti-PD-1 or anti-CTLA-4 antibodies. Advanced delivery techniques are improving the efficacy of ICIs by enhancing targeting and reducing side effects. Exosome-based systems transport immune-modulating molecules naturally, while injectable hydrogels provide localized, sustained drug release. Microneedle patches enable transdermal ICI delivery, and bioconjugation enhances specificity to the TME. Combination therapies with cytokines or chemotherapeutics further boost immune responses and therapeutic outcomes [104].

## Engineered chimeric antigen receptor (CAR)-T cell therapy (a “living drug”)

**Figure 3:**
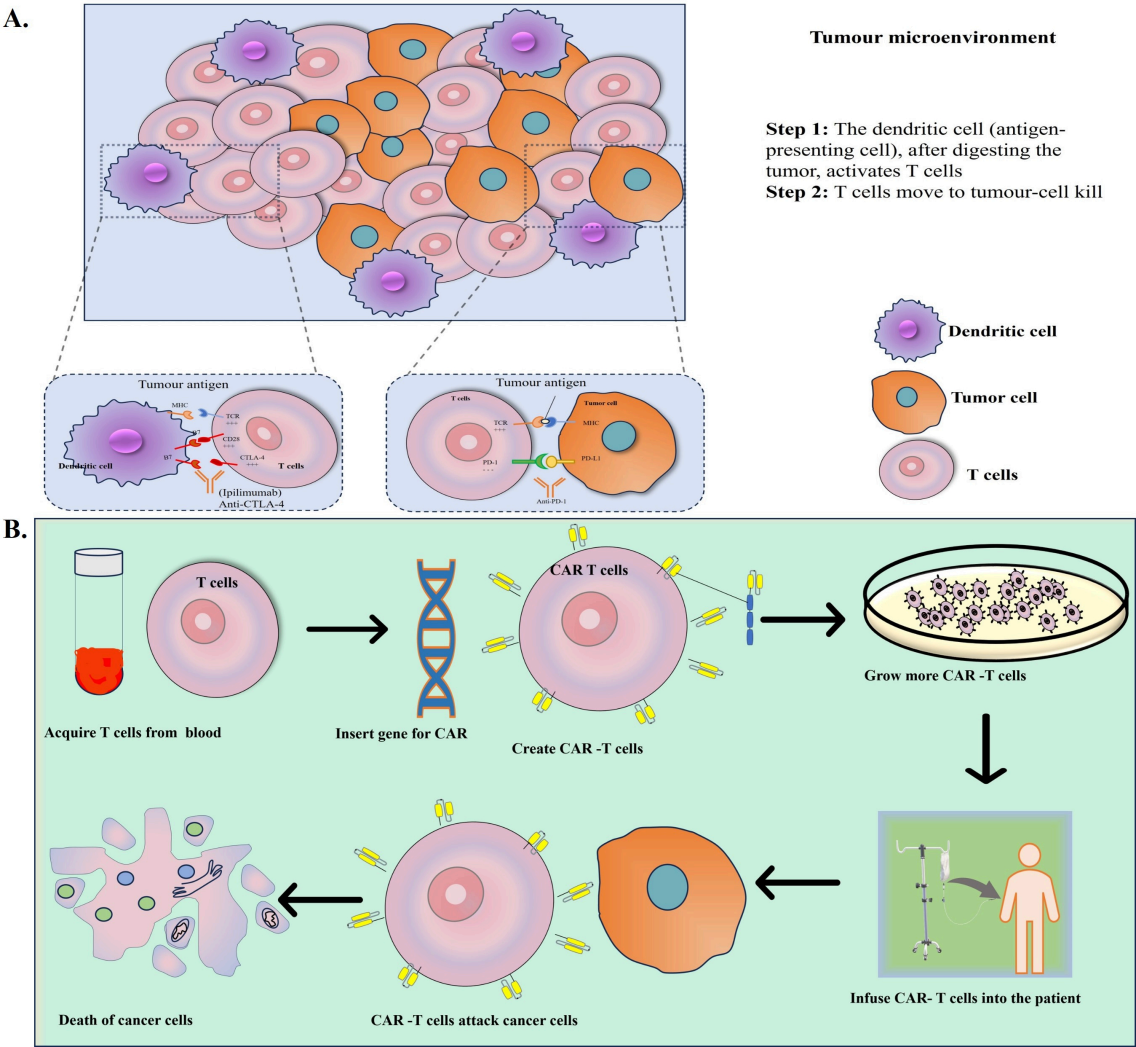
**(A) Cells of tumor microenvironment, (B) simplified steps of CAR-T cell infusion into the patient**. Further graphic representation of death of cancer cells post-infusion of CAR-T cells [240]. CAR: chimeric antigen receptor *Note.* Adapted from “Nanomaterials in tumor immunotherapy: new strategies and challenges” by Zhu X, Li S. Mol Cancer. 2023;22:94 (https://molecular-cancer.biomedcentral.com/articles/10.1186/s12943-023-01797-9). CC BY.

**Figure 4:**
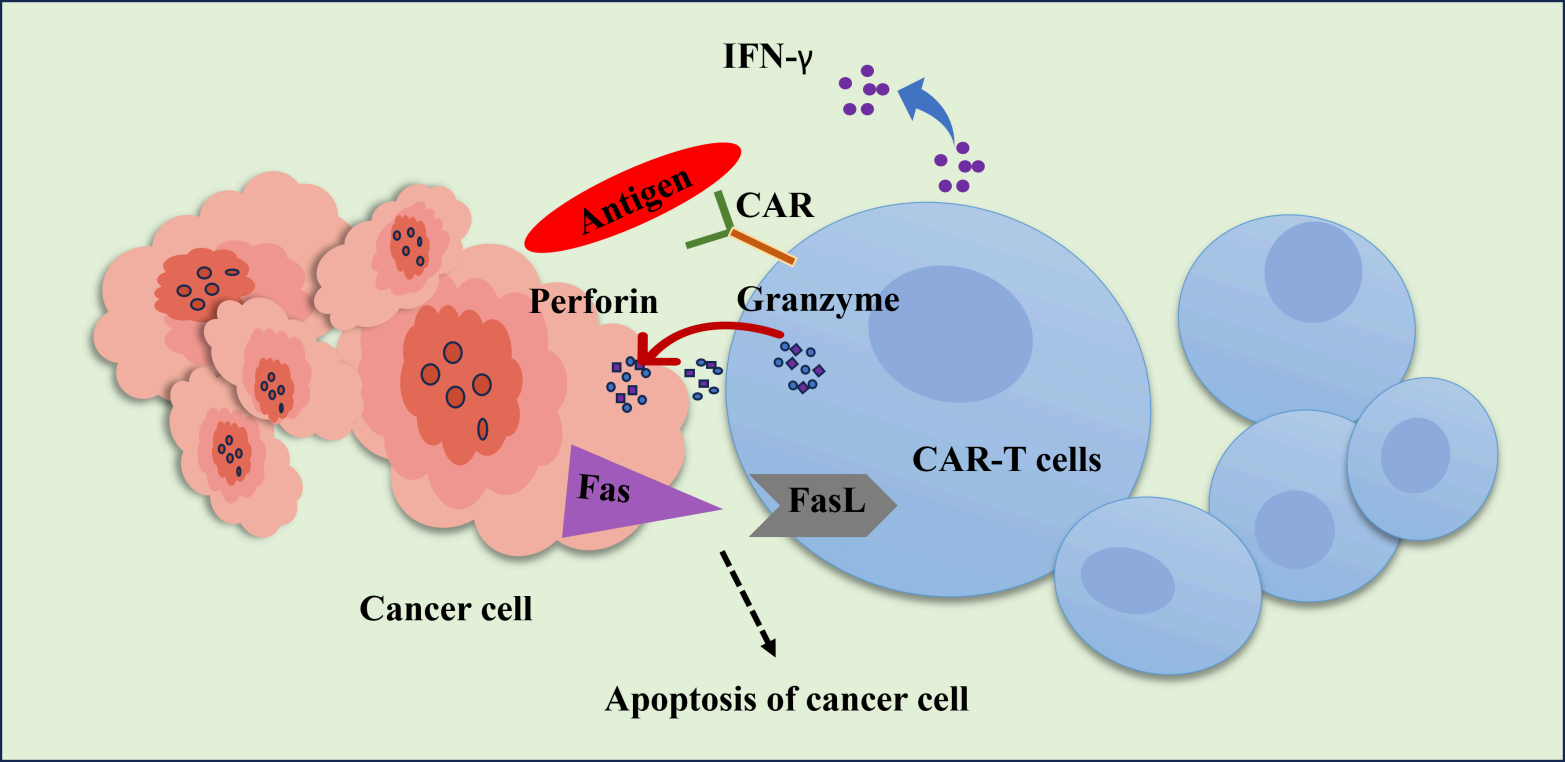
**Schematic representation of the CAR-T cell’s cytotoxic action against cancer cells**. To exert anti-tumor action, activated CAR-T cells can selectively detect the tumor antigen and secrete granzyme, perforin, and IFN-γ. The death receptor pathway through Fas/FasL mediates CAR-T cells’ anti-tumor activity and triggers the death of cancer T cells. FasL: Fas ligand; IFN-γ: interferon-gamma; CAR: chimeric antigen receptor

An intriguing advancement in cancer immunology is chimeric antigen receptor (CAR)-T cell treatment, in which patient immunological T cells are extracted, modified to produce “CAR”-T cells, and then reinfused into the same patient (Figure 3). CAR is a recombinant receptor construct that allows T-cell-mediated cytotoxicity to be redirected to cancer cells in an HLA-independent way by attaching an extracellular single-chain variable fragment (scFv) produced from an antibody to intracellular T-cell-signaling domains of the T-cell receptor [108]. CAR-T cells are one of these immunotherapeutic strategies that have demonstrated exceptional usefulness in treating hematological malignancies, such as acute lymphoblastic leukemia, myeloma, and non-Hodgkin’s lymphoma, US FDA has approved these uses [109]. CAR-T cells release perforin, granzyme, and IFN-γ to directly combat antigen-positive tumor cells (Figure 4). Additionally, antigen-negative tumor cells, trigger apoptosis through death receptor ligands like Fas ligand (FasL) [110].

The first CAR-T cell therapy approved by the US FDA in 2017 was Kymriah^®^ (an anti-CD19 CAR-T), which targets B-cell malignancies. Apart from this, Yescarta, Tecartus, Breyanzi, Abecma, and Carvykti were gradually developed, which also target anti-CD19 CAR-T cells, whereas, Abecma and Carvykti target B-cell maturation antigen (BCMA). Leukapheresis is used to prepare the patient’s autologous CAR-T cells, which load a CAR that targets BCMA for multiple myeloma and CD19 for B-cell malignancies [96]. Replication incompetent retroviruses (for Yescarta and Tecartus) or lentiviruses (for everyone else) were used to transduce the CAR-T transgene into the cells. It also contained a co-stimulatory molecule (CD28 for Yescarta and Tecartus and CD137, also called 4-1BB, for everyone else). The CAR transgene was transduced into cells using replication-incompetent retroviruses (for Yescarta and Tecartus) and lentiviruses (Breyanzi, Abecma, Carvykti, Kymriah) [111]. All these are used for hematological malignancies, including lymphomas, leukemia, and multiple myeloma [111]. However, these approved CAR-T cell products share the adverse events of cytokine release system (CRS), like immune effector-cell associated neurotoxicity syndrome (ICANS), cytopenia, and hypogammaglobulinemia. Currently, investigational approaches are focused on further potentiating the efficacy of CAR-T cells in non-responding patients and solid tumors [112]. The physical barriers of solid tumors, such as the tumor stroma, also restrict CAR-T cells’ ability to traffic, penetrate, and infiltrate them [113].

### NP-based CAR-T therapy

In a recent phase III clinical trial, epacadostat (EPA), the most sophisticated IDO1 inhibitor, failed to treat metastatic melanoma when used in conjunction with a PD-1 checkpoint inhibitor. An EPA nanovesicle therapeutic platform (Epacasome) based on chemically binding EPA to sphingomyelin was reported by Wang et al. [114]. Compared to free EPA, epacasome exhibits greater cellular absorption through clathrin-mediated endocytosis, as well as improved T cell proliferation and IDO1 inhibition. With deep tumor penetration and effective intratumor drug release, epacasome exhibited enhanced PKs and tumor accumulation in a B16-F10 melanoma model, and provided better anticancer activity, enhancing PD-1 blockage with increased CTLs and decreased Tregs and MDSCs than free EPA. Epacasome further improves anti-tumor effects and immune responses by co-encapsulating immunogenic dacarbazine. This is especially true when paired with the PD-1 inhibitor in the late-stage metastatic B16-F10-Luc2 model in female mice, which upregulates NKG2D-mediated CTLs and NK cell responses. This combination also prolongs animal survival and reduces tumor recurrence in a therapeutically relevant post-surgical melanoma model in female mice [114].

For T cell transfection and differentiation, the activation stage is essential and necessitates the involvement of CD3/TCR and CD28. While APCs aid in in-vivo activation, antibodies against CD3 and CD28 linked to magnetic beads are required for ex-vivo activation. Although this artificial activation works well, before clinical use the beads need to be removed, which makes the process of producing CAR T cells more difficult. Combining the transfection properties of LNPs with the activation of magnetic beads, activating lipid NPs (aLNPs) imitates APCs. In a mouse xenograft model, it is demonstrated that aLNPs allow for the one-step activation and transfection of primary human T cells, with the resultant mRNA CAR-T cells lowering tumor burden. This confirms that aLNPs are a promising platform for the quick generation of mRNA CAR-T cells [115].

## CAR-macrophages, potential alternative for CAR-based solid tumor immunotherapy

The implementation of CAR-T cell treatment in solid tumors has been difficult due to T cells’ low ability to infiltrate and survive in the TME, despite its demonstrated effectiveness in hematologic malignancies [116]. Researchers have looked into macrophages as potential candidates for the next CAR platform to get over these restrictions because of their ability to be the most prevalent and deeply infiltrated into the solid tumor TME. Macrophages target and destroy both aberrant and infected cells, making them a key part of the innate immune response [117]. To stimulate adaptive immunity, they also deliver antigens to T cells and demonstrate phagocytic activity against malignancies [118]. Additionally, the solid tumor immunosuppressive TME is modulated and remodeled by macrophages through the secretion of cytokines and chemokines [118]. They are classified as M1 or M2 macrophages based on their phenotypic and functional traits [118]. CAR-macrophages (CAR-M) therapy is a promising approach involving genetically engineered macrophages. Innate immune cells known as TAMs are primarily M2 macrophages with a minor fraction of M1 macrophages [119]. Macrophages enter solid tumors and change to the pro-tumor M2 subtype responding to chemokines and growth factors released by cancer cells [120]. The most prevalent immune cells in the TME of solid tumors, TAMs are essential to the progression of the tumor. In many solid tumor forms, TAM infiltration is clinically associated with a poor prognosis [119]. TAMs are implicated in numerous aspects of tumor progression, such as immune suppression, tumor metastasis, and cancer cell proliferation, researchers are interested in directly targeting them for therapeutic approaches to increase the effectiveness of cancer treatments [121]. TAMs have limited anti-tumor actions due to their complex flexibility and heterogeneity [74]. CAR-T, CAR-M’s fundamental structure consists of an intracellular domain that triggers downstream signaling pathways, a transmembrane domain, and an extracellular antigen recognition domain known as scFv [122]. Antigen-expressing tumor cells trigger the cytotoxicity of CAR-M, and CAR-M activation modifies TME [123]. When at M0 condition it is exposed to a tumor antigen, CAR-M transforms into an M1 pro-inflammatory phenotype and has an anti-tumor impact [117]. In the TME, this activated CAR-M stimulates innate immune cells and releases pro-inflammatory cytokines. CAR-M’s phagocytic activity selectively identifies and kills tumor cells. By presenting antigens and triggering T-cell cytotoxicity, CAR-Ms can also strengthen the adaptive immune system to produce synergistic anti-tumor effects [124]. When M1 macrophages enter the TME of solid tumors, CAR-M cells phagocytose them, demonstrating their potent anti-tumor activity [117]. However, to engineer CAR-M cells, a differentiated M1 phenotype is necessary [125].

### NP-based CAR-M therapy

CAR-M treatment is now being developed using a range of nanobiomaterials, such as LNP formulations, cationic polymers, and biocompatible hydrogels, due to the aforementioned special qualities and benefits. These materials have a lot of promise for in vivo CAR-M treatment approaches and can be used as substitutes for viral vectors in the delivery of CAR genes [126]. CAR macrophages and T lymphocytes can be engineered in vitro via lipid NP-mediated mRNA delivery. A study demonstrated the great potential of LNP-mRNA technology by performing mRNA transfection on T-cells and macrophages for adoptive cell therapies. The resultant CAR-T and CAR-M cells demonstrated a significant cytotoxic effect on B lymphoma in vitro [127]. To generate CAR-T cells for targeted transfection Zhou and colleagues [128] created an LNP system with a modified CD3 antibody. By delivering a combinatorial gene of CD19 CAR and IL-6 short hairpin RNA (IL-6 shRNA), they were able to convert T-cells into IL-6 downregulated CAR-T cells, which eliminated leukemic tumor cells with high CD19 expression while lowering the CRS brought on by IL-6 [128]. Both these CAR-M and CAR-T cell-based production ways of employing LNPs following the conventional route procedure were lengthy as well as very costly, and required cellular modification in adhering to strict manufacturing quality management criteria. Which new generation of CAR-M technology has been developed using the acquisition of CAR-M in vivo via non-viral vectors. Kang et al. [129] tried to deliver the combinatorial gene encoding CAR and IFN-γ into macrophages in situ using macrophage-targeted polymer nanocarriers (MPEI/pCARIFN-γ). After implantation of CAR-encoded plasmid DNA nanocomposites and macrophage-targeting nanocarriers, tumor-bearing mice developed CAR-M1 macrophages, which facilitated tumor phagocytosis, anti-tumor immunomodulation, and inhibited solid tumor growth. With additional support from cytokines, this approach enhanced the immunomodulatory and tumorigenic potential of CAR-M products [129]. An injectable hydrogel “drug reservoir” device was created to deliver CD47 antibodies and macrophage-targeted altered nanocarriers (pCAR-NPs) in a “filled form” to the postoperative tumor cavity of GBM. pCAR-NPs work in situ on the “local” macrophage surrounding the postoperative tumor cavity, producing CAR-M there that targets the removal of glioma stem cells (GSCs); in the meantime, CD47 antibodies prevent tumors from sending the “do not eat me” signal. Consequent utilization of its antigen presentation effect concomitantly activates the adaptive immune system and increases the phagocytic efficacy of CAR-M against GSCs. By eliciting the immunological memory effect after treatment, these synergistic reactions prevented glioma recurrence. The high expenses and drawn-out procedure associated with conventional CAR-cell production could thus be avoided by using the nanobiomaterials vector in vivo transfection approach, which also avoids the safety issues brought on by viral vectors in vivo [127].

## CAR-NK cell therapy

NK cells, the innate immune cells that are CD3-negative and CD56-positive consist of around 5–15% of human peripheral blood mononuclear cells (PBMC) [130]. The initial line of defense against cancers and viral infections, they function as effector cells, are independent of tumor antigens, and have no memory [131]. Additionally, they control the death of cancer cells by recognizing target ligands in a pattern [132]. NK cells can be widely triggered by their activating and inhibitory receptors, while CAR-T cells can only target tumor cells utilizing particular antibodies against scFv [133]. The regulation of NK cells’ cytotoxic activity depends on the balance between their activating and inhibitory receptors. The NK cells’ activating receptors, including NKp46, NKG2D, DNAX accessory molecule-1 (DNAM-1), NKp44, and NKp30 cause NK cells to release granzyme B and perforin, which kill tumor cells [130]. Activated NK cells can cause targeted cell death by using the death receptor pathway [134]. NK cells expressed death ligands on their surface attach themselves to the target cancer cells’ death receptor which in turn induces death of the cancer cells. Death ligands like FasL and/or TRAIL are expressed by NK cells [135]. Numerous sources, such as peripheral and cord blood, induced pluripotent stem cells (iPSCs), and cell lines, can produce CAR-NK cells [136]. Both ADCC-independent and CAR-dependent mechanisms can control the tumor-killing capacity of CAR-NK cells [137]. CAR-NK cells can be used in “off-the-shelf” allogeneic therapy since they are less dangerous than CAR-T cells and have a lower risk of CRS, neurotoxicity, and Graft-versus-host disease (GvHD) [137]. Despite its apparent advantages over CAR-T cells, CAR-NK cells still face significant restrictions and challenges. Solid tumors are challenging for CAR-NK cells to penetrate due to tumor heterogeneity and immunosuppressive TME [130].

### NP-based CAR-NK therapy

Nanotechnology offers a substitute for traditional CAR-T treatment. Recent studies have attempted to achieve CAR-NK with enhanced efficiency by increasing transfection efficacy using NPs [138, 139]. McKinlay et al. [140] created the charge-altering releasable transporter for successful mRNA delivery. At low pH, the carbonate-*b*-α-amino ester is cationic; however, at pH 7.4, it undergoes a rearrangement. Because of these characteristics, oligomers can secure and transport polyanionic molecules, like mRNA, inside cells by forming complexes with them at low pH levels. Following this, the liberated mRNA is translated into proteins, and the oligomers undergo biological degradation [140]. PEI-coated magnetic NPs (MF-NPs), having a magnetic core (Zn/Fe) have also been developed for multifunctional application in CAR-NK treatment. The magnetic core is designed for in vivo tracking and magnetic resonance imaging (MRI), while the PEI shell offers an electrostatic attraction for anti-EGFR CAR pDNA to bind, transfecting NK cells. Through endocytosis, NK-92MI cells are internalized MFNP/anti-EGFR CAR pDNA, which demonstrated a noteworthy degree of in vitro transfection efficiency, illustrating no particular toxicity to NK cells with a strong antitumor impact [141]. Efficiency and stability were better than those of the viral vector or EP. The biological behavior of CAR-NK cells could be observed by near-infrared radiation (NIR) fluorescent dye (cyanine 7) attached to the PEI shell, and in vivo may be seen using both MRI and a fluorescent imaging device. Therefore, the use of this multifunctional NP may help streamline and effectively change the CAR-NK therapy procedure [141].

## Recompenses of NPs for immunotherapy

Nanotechnology advancements have made nanocarriers a promising drug delivery method for effective cancer therapy [142–146]. These benefits include: easy modification of biology-active moieties on the surface for tumoral biomarker recognition; rational size, structure, and morphological design; spatiotemporal control in multi-functions; reduced side effects; flexibility to combine other synergistic therapies; and targeted and controlled drug release in tumor sites [147–149]. However, a major obstacle is posed by the body’s numerous physiological barriers, which prevent the effectiveness of therapy (Figure 1). These challenges in tumor treatment have prompted the development of methods for more precisely and less invasively targeting the tumor spot [150]. The unique characteristics of bioactive NPs, including size, shape, charge, flexibility, and carrier functionality, make them the preferred choice for immunotherapy [75].

Nano-immunotherapy is increasingly being used to treat cancer. Nano-immunotherapy is a highly interdisciplinary approach that integrates nanotechnology, immunology, and oncology to enhance cancer treatment. By combining the precision of nanotechnology for targeted drug delivery with immunological insights into tumor immunology and the latest cancer therapies, this integrated strategy allows for more effective immune activation and tumor targeting. Emphasizing this collaboration between fields highlights the innovative potential of nano-immunotherapy, showcasing how advancements in each discipline contribute to improving treatment outcomes and overcoming current therapeutic challenges. Through a range of techniques, nano-immunotherapy can boost defenses against cancer. Therapeutic medicines can be precisely administered to the targeted spot by using NPs that have been tailored to target certain cells or tissues. NPs can govern the steady release of immunotherapeutics, exposing immune cells to the active components for prolonged periods. This extended exposure may prolong the maintenance of therapeutic levels, thereby increasing the treatment’s effectiveness. Surface functionalization of ligands that bind to overexpressed receptors on tumor cells or APCs can accomplish better efficacy [151]. By acting as transporters for adjuvants and antigens, NPs can facilitate the absorption of these substances by APCs like DCs. NPs can boost strong T-cell responses by improving antigen presentation, which is essential for successful immunotherapy [152]. Some NPs have inherent immunomodulatory characteristics that allow them to either stimulate or inhibit immune responses. This trait is especially useful for adjusting the immunological milieu in a way that promotes immunity against tumors [153]. NPs have the potential to alter the immunosuppressive environment in TME by targeting its primary components. Hypoxia is a direct result of the distorted blood vessels in TME and the fast growth of tumor cells. This leads to the accumulation of immunosuppressive cells, such as Tregs and MDSCs, as well as the secretion of immunosuppressive factors, such as VEGF and TGF-β. These replacements cause aberrant fibrosis, shift macrophages to the pro-tumorigenic M2 phenotype, and impair DC (immature DC; iDC) activities [154].

Nano-immunotherapy plays a dual role in the advancement of cancer treatment, functioning both as an enhancer of the immune system and as an independent therapeutic agent. As an enhancer, it amplifies the body’s existing immune responses, improving the efficiency and effectiveness of traditional treatments such as immunotherapies and vaccines. Simultaneously, its potential as a standalone therapeutic agent lies in its ability to directly target cancer cells with precision, leveraging nanoscale technologies to deliver drugs, modulate the TME, and stimulate immune activity. This dual functionality underscores the transformative potential of nano-immunotherapy in modern oncology [155]. Additionally, the size and surface charge influence cellular uptake and biodistribution, ensuring that NPs accumulate at the tumor site and reduce off-target effects. Functionalization with specific ligands or antibodies allows NPs to target immune checkpoints like PD-1/PD-L1, or MDSCs, which are critical in immune evasion. By selectively modulating these specific immune components, nanotechnology enhances the anti-tumor immune response, overcoming the immune suppression mechanisms that tumors often exploit to evade detection. These innovations lead to improved therapeutic outcomes, with increased tumor penetration, prolonged drug release, and reduced toxicity, making nanotechnology a powerful tool in cancer immunotherapy [155]. The synthesis and physicochemical characterization of thermoresponsive nanogels based on poly(*N*-isopropylacrylamide) (pNIPAM) and their in vitro, ex vivo, and in vivo (mice model) performance. Therefore, we have demonstrated that pNIPAM nanogels can be used as an efficient platform for vaccine nanocarriers. Evaluate pNIPAM nanogels cytotoxicity was performed in different cell lines showing high biocompatibility (> 70%). Using the outer membrane lipoprotein A (OmlA), an important virulence factor of porcine pleuropneumonia *Actinobacillus pleuropneumoniae* (App) was used to deliver and protect antigens. The biodistribution of pNIPAM nanogels was administered intranasally and showed the presence in the lungs during the evaluated time. BALB/c mice injected with OmlA encapsulated into pNIPAM nanogels showed higher antibody titres than those of OmlA with aluminum hydroxide adjuvant. The outcomes demonstrated that nanogels could elicit a humoral immune response [156].

## Microneedle-based drug delivery

**Figure 5:**
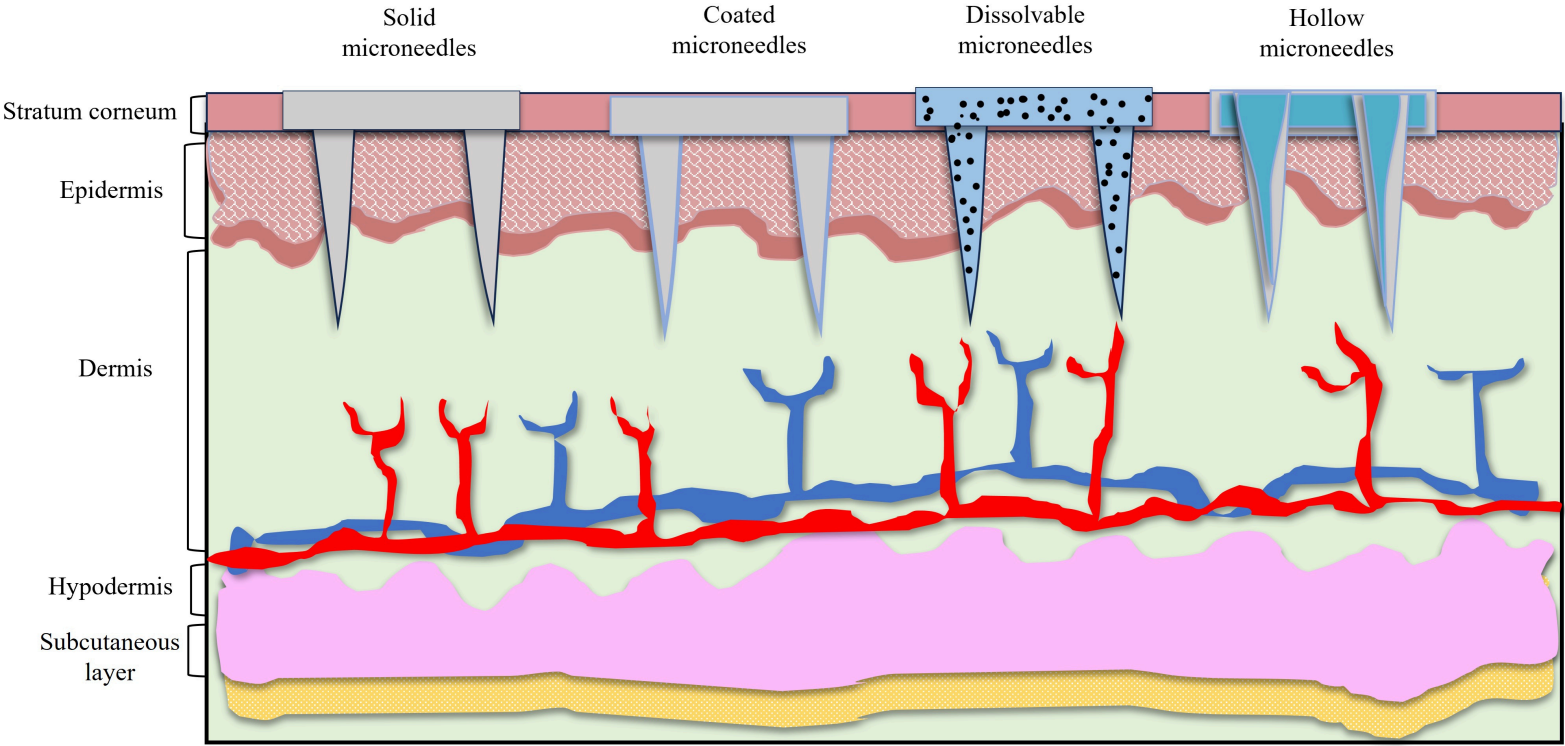
Types of microneedles for transdermal administrative mode, demonstrative images of solid microneedles, coated microneedles, dissolvable microneedles and hollow microneedles

One promising technique for administering immunotherapeutics is transdermal administration. Dissolving microneedles, mostly composed of soluble or biodegradable polymers, have attracted a lot of interest because of their outstanding drug loading capacity, ease of availability, painlessness, safety, and convenience, which makes them perfect transdermal delivery system (Figure 5). Through this dissolving microneedles ICIs, cancer vaccines, and adoptive cell treatment can be delivered for their potential clinical translation [157]. MNs are the most frequent devices implemented in transdermal immunotherapy of cancers (e.g., melanoma, squamous cell carcinoma, cervical, and breast cancer), as well as other infectious diseases. As a new therapeutic strategy for the treatment of cancer, transcutaneous vaccines can provide therapeutic benefits in cancer immunotherapy. MNs can aid in delivering cancer vaccinations to dermal immune cells in a painless manner. The ability of MNs to administer cancer vaccines was shown in several preclinical investigations [158].

He et al. [159] used 2-(diisopropylamino)ethyl methacrylate-b-methacrylic acid (PDM, a copolymer) in the structure of coated MNs to reduce the application time to one minute. PDM is a polymer that is charge-invertible due to pH. Consequently, in comparison to animals given intramuscular and subcutaneous injections, the MN system dramatically raised the OVA-specific IgG1 level and sustained antigen (OVA) release for three days. Significant antigen absorption was also shown by the human APCs on skin tissue [159]. In a related study, Lee et al. [160] employed the OVA antigen to trigger an immunological response in a mouse model. MNs loaded with soluble OVA enhanced the number of OVA-specific CD8^+^ and CD4^+^ T lymphocytes and efficiently eliminated EG7 tumor cells that expressed ovalbumin. In mice that received the vaccination, it also prevented tumor development and angiogenesis [160]. In a study designed, dissolvable MNs were loaded with a liposome containing OVA antigen and platycodin, a saponin adjuvant. Liposomes decreased platycodin toxicity and increased OVA absorption by mouse bone marrow DC. Equivalent Th1 was created by platycodin, and humoral immunity was triggered by Th2. When administered to mice, MNs considerably enhance their immune response to OVA and cause very minor cutaneous irritation in rabbits [161].

Dissolvable MN effectiveness was evaluated on melanoma mouse models. By triggering an immunogenic response against antigens, these dissolvable MNs can improve immune cells’ recall memory and accelerate the removal of cancer cells from lung tissue [162]. The microneedle cocktails comprising a bioresorbable polypeptide matrix with a nanopolyplex, having cationic amphiphilic conjugates with ovalbumin-expressing plasmid OVA (pOVA) and immunostimulant-polyinosinic-polycytidylic acid [poly(I:C)]. The pOVA and poly(I:C) were effectively transported into the intracellular compartments of DCs and macrophages.

The therapeutic effect on B16/OVA melanoma tumors was enhanced by the dissolving microneedle cocktail therapy, which enhanced the therapeutic efficacy. Remarkably, the cocktail-based therapeutic vaccination also led to improved lung clearance of cancer cells and improved antibody recall memory after challenge compared to standard vaccination [162]. Another study on melanoma, a core-shell MNs system (CSMN) was created for the tropical transformation of [1-methyl-*D*, *L*-tryptophan (1-MT), a checkpoint inhibitor, and anti-PD-1/PD-L1 antibody (aPD-1/aPD-L1)]. The premature crystallization was prevented with an increased amount of 1-MT, thereby facilitating the PD-L1 in MN tips, which in turn imposes sustained release activity of PD-L1 for improved drug delivery efficacy [163]. Melanin-loaded polymeric MNs were created to stimulate anticancer activity in skin DC using the B16F10 melanoma mouse model. The patch contains granulocyte monocyte colony-stimulating factor (GM-CSF), B16F10 whole tumor lysate, and melanin, a natural pigment applied as a photosensitizer agents. Melanin converts light into local heat through the emission of NIR light, thereby improving immunologic responses, and releasing proinflammatory cytokines and danger signals (e.g., TNF-α, IL-6, IFN-γ, HSP70, and HSP90 expression), immune cell recruitment, increased lymphatic or blood flow at the execution site. Furthermore, the MNs successfully delivered the cancer vaccine to the target cells in the epidermis, and the majority of the vaccinated mice showed good tumor rejection and a long survival rate [164]. Increasing response rates and overcoming drug resistance are now the main obstacles facing cancer immunotherapy. Since the skin is a highly active immune organ with a huge population of resident APCs, dermal injection proves to be a potential immunotherapy delivery method. The epidermis is rich in immune cells, and microneedle arrays can penetrate it to trigger a strong T-cell response in the tumor cell microenvironment [165].

Microneedle patch loaded with pH-responsive tumor-targeted lipid NPs (NPs), which permits local delivery of aPD-1 and cisplatin (CDDP) precisely to cancer tissues for cancer therapy. The aPD-1/CDDP@NPs administered using microneedles significantly increased the immune response for in vivo experiments, which led to a notable influence on tumor regression. Consequently, a strong microneedle-induced T-cell response, aPD-1-mediated T-cell PD-1 blockade, and increased CDDP direct cytotoxicity in tumor cells all triggered synergistic anti-cancer processes. The animal model that was not responding to aPD-1 systemic therapy showed a remarkable increase in response rate when transdermal distribution utilizing MNs was used. In the treatment of malignancies that do not respond to immunotherapy, this showed promise [166]. Photodynamic treatment and transdermal immunotherapy were employed to treat breast cancer in mice. Zinc phthalocyanine, a photosensitizer, and an anti-CTLA-4 antibody were co-delivered using MNs. When immunotherapy and photodynamic treatment were used together tumor growth inhibition was more effective when they were used individually. Additionally, it successfully stimulated the cytotoxic T cell response and activated CD3, CD4, and CD8 positive T cells, in contrast to immunotherapy and photodynamic therapy [167].

Lipid-polymer conjugate-based amphiphilic vaccines are a novel class of vaccination that can self-deliver to the immune system. Amphiphilic vaccines effectively target APCs in the lymph nodes through a special albumin-mediated transport and uptake mechanism when administered subcutaneously. They also elicit strong humoral and cellular immune responses. For which, a study was conducted to investigate the efficiency of MNs in administering amphiphilic vaccinations. To trigger an immunological response, MNs target APCs. When mice were immunized with amph-OVA323–339 and amph-CpG, their serum antigen-specific IgG and IFN-γ-producing CD4^+^ T cells increased. This suggests that dissolving MNs increased the efficiency of the amphiphilic peptide vaccine in eliciting humoral and cellular responses in the mouse model [124].

In a different DNA vaccination experiment cationic RALA/pDNA NPs were used to target prostate cancer cells to assess the effectiveness of a two-tier delivery in a dissolvable MN patch. Application of NP-loaded MN patches successfully resulted in endogenous production of the encoded prostate stem cell antigen (PSCA). Additionally, ex vivo vaccination with MNs loaded with RALA/pPSCA induced a tumor-specific immune response against TRAMP-C1 tumors. In vivo, vaccination with RALA/pPSCA-loaded MNs showed anti-tumor efficacy in both therapeutic and prophylactic prostate cancer models and was restricted to vaccinated animals [168]. Ali et al. [169] used a restricted patch to assess the effectiveness of the HPV-16 E6/E7 DNA vaccination in treating cervical cancer. A peptide called RALA was used in the vaccination to compress the E6/E7 DNA in cationic NPs and PVP MNs skin penetration. In the MNs vaccinated group, MN/RALA-E6/E7 increased E6/E7-specific IgGs and IFN-γ production, which in turn increased humoral response and T cell-mediated cellular cytotoxicity. Additionally, it inhibited the growth and development of tumors in the therapeutic and prophylactic models, respectively [169]. An amphiphilic triblock copolymer-based dissolving MN was created which forms nanomicelles inside after penetration. It’s able to deliver encapsulated poorly water-soluble TLR7/8 agonist (R848) and other hydrophilic antigens after cutaneous application. Significant anticancer efficacy was produced by applying MNs containing tumor model antigen (OVA) and R848 to the skin of EG7-OVA tumor-bearing mice. This application caused a high degree of antigen-specific humoral and cellular immunity [170].

## Adjuvants nanomaterials/NPs for TME

**Figure 6:**
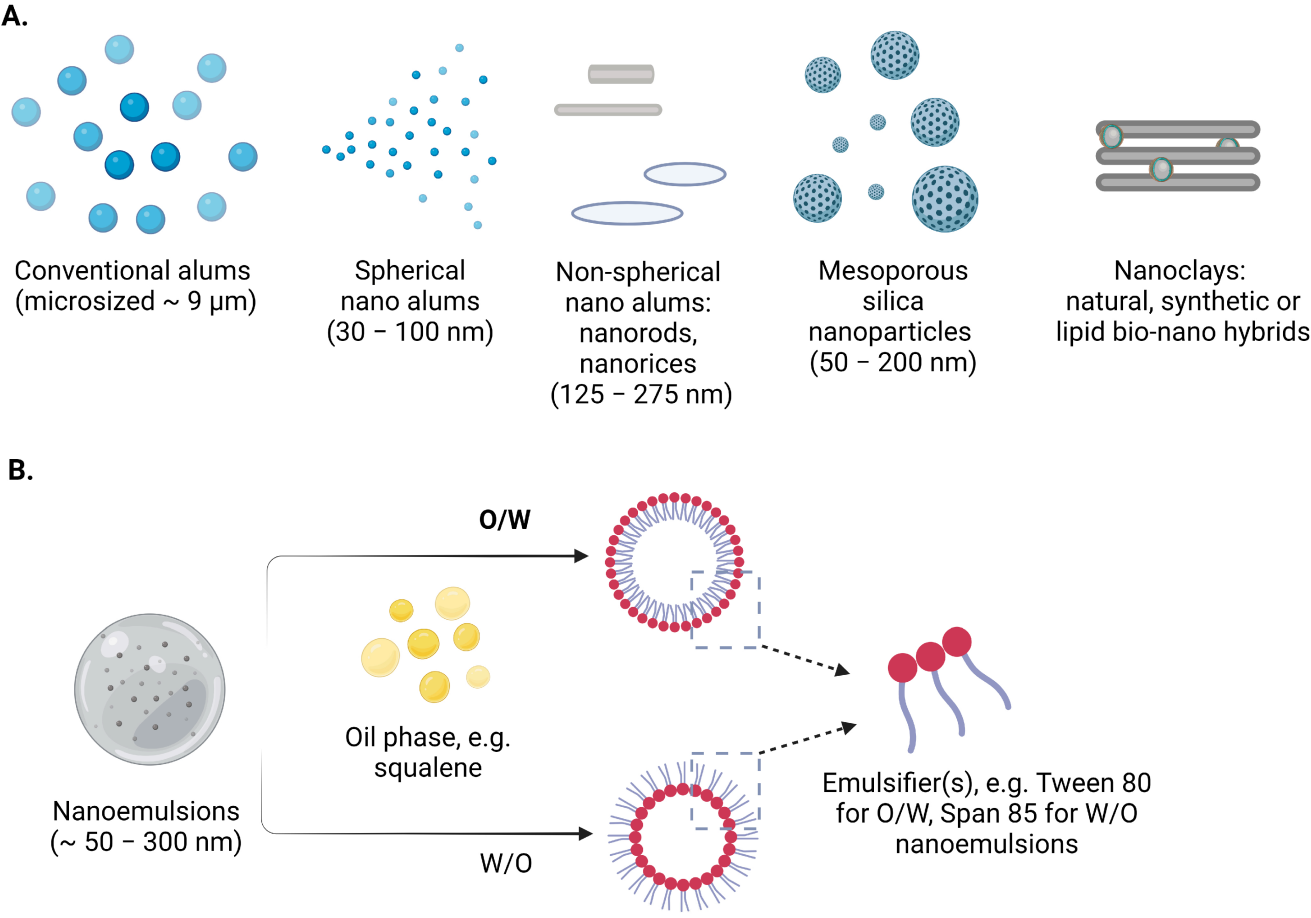
Schematic representation of the selected (A) inorganic nanomaterials (micro- to nano- sized) and (B) nanoemulsions as adjuvants and/or carriers in contemporary vaccine formulations *Note.* Reprinted from “Nanoparticle-Based Adjuvants and Delivery Systems for Modern Vaccines” by Filipić B, Pantelić I, Nikolić I, Majhen D, Stojić-Vukanić Z, Savić S, et al. Vaccines (Basel). 2023;11:1172 (https://www.mdpi.com/2076-393X/11/7/1172). CC BY.

Multifunctional inorganic nanomaterials also have gained significant attention in the biomedical area during the past 20 years due to their potential in drug delivery, tumor treatment, and imaging [58, 102, 171]. Certain inorganic NPs stimulate the immune system by encouraging the growth and activation of immune cells. The adjuvant activity of these nanomaterials can be influenced by their charge, size, shape, and composition. As a result, inorganic nano adjuvants often fall within a certain size and form range [172]. Adjuvants containing aluminum (Alums) are the most often used and usually considered safe adjuvants and have been used as immunostimulants in vaccinations [173]. The approved vaccines contain several aluminum compounds, including aluminum hydroxide, aluminum phosphate, and amorphous aluminum hydroxyphosphate sulfate [174]. However, their unique physicochemical characteristics may have a significant impact on their immunomodulatory effects [175]. The primary NPs of the aluminum adjuvants are fibers that form loosely linked porous aggregates, including conventional alums, spherical nanoalums, mesoporous silica NPs (MSNs), nanoclays, nanoemulsions, etc. (Figure 6) [102]. These aggregates serve as the functional units of vaccines, and particle size, charge, and isoelectric point (IEP) vary from 4.6 to 11.1 of aluminum adjuvants, depending on the salt, resulting in different charges in the physiological environment [176]. These charges can be very significant for the interaction with the antigen, and can also differ significantly [102]. The layered double hydroxide and hectorite clay NPs, also known as nanoclays, demonstrated robust adjuvant activity that produced immunological responses that were noticeably more powerful than those produced by commercial adjuvants [102]. Inorganic materials have been extensively studied as vaccine adjuvants due to their ability to be synthesized at the nanoscale and have their structural and functional properly finely controlled and promote the prolonged and targeted release of antigens, thereby increasing immunogenicity, triggering immune response [177, 178]. Furthermore, the Th1-type cellular immune response was strongly stimulated by aluminum hydroxide NPs containing the EsxV antigen of *Mycobacterium tuberculosis*, which makes it an effective adjuvant against *M. tuberculosis* infection [179]. Investigations were also conducted into the effects of aluminum adjuvant surface coatings. Phospholipid bilayer-coated aluminum NPs (PLANs) when coated with adjuvants demonstrated acceptable stability. Furthermore, coated adjuvants were more efficiently absorbed by APCs, eliciting potent antigen-specific humoral and cellular immune responses with reduced local inflammation [180].

MSNs are possible due to their intrinsic structural properties, such as large pore volume, high specific surface area, low density, good biocompatibility, thermal and chemical stability, and ease of chemical functionalization. MSNs have been demonstrated to successfully improve both humoral and cellular immunity in animal models when used as an immunological adjuvant. Specifically, intramuscular or oral administration of bovine serum albumin (BSA) encapsulated/adsorbed SBA-15 nanostructured silica increased immunogenicity and stimulated mutually Th1 and Th2 immune responses in mice, whereas intraperitoneal injection of ovalbumin and amorphous silica NPs has a supplemental impact on Th1, Th2, and Th17 immune responses [102].

**Figure 7:**
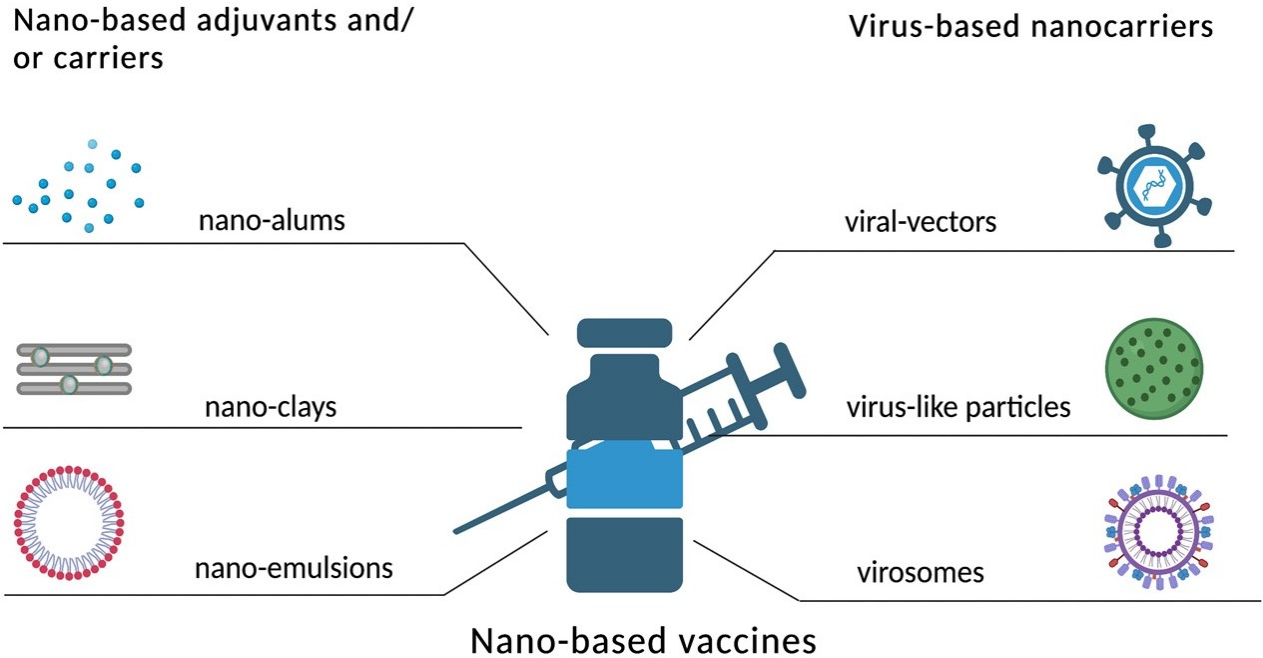
Representation of nano-based adjuvants and virus-based nano-carriers *Note.* Reprinted from “Nanoparticle-Based Adjuvants and Delivery Systems for Modern Vaccines” by Filipić B, Pantelić I, Nikolić I, Majhen D, Stojić-Vukanić Z, Savić S, et al. Vaccines (Basel). 2023;11:1172 (https://www.mdpi.com/2076-393X/11/7/1172). CC BY.

Solid lipid NPs and liposomes are examples of lipid-based NPs. They can help deliver antigens to APCs in a specific manner and encapsulate both hydrophilic and hydrophobic substances. Liposomal vaccines have been demonstrated in studies to improve T cell activation and DC uptake. Explores a novel liposomal vaccine designed to target DCs, significantly improving the delivery of antigens and leading to enhanced T-cell activation. The study demonstrates that this targeted approach results in a stronger immune response, suggesting the potential for more effective vaccines in cancer immunotherapy and infectious diseases. In a study, based on viral antigen sequence, mRNA-based vaccines are designed and manufactured on a clinical scale for a week. However, obstacles were faced during the development of mRNA-based vaccines using nanodelivery systems, such as the high molecular weight of mRNA, negatively charged mRNA, intrinsic instability, and high susceptibility to degradation by ribonuclease [181]. The nano-carriers of NP vaccines, which are next-generation vaccination technologies, include liposomes, polymers, inorganic NPs, particles that resemble viruses, and self-assembling protein NPs. Immune cells can more easily recognize and digest these cleverly designed NP vaccinations to produce enhanced innate and adaptive immune responses (Figure 7). Therefore, nanodelivery systems are crucial for the successful in vivo delivery of mRNA to the site of action. Moreover, the controlled release of antigens can be achieved by polymeric NPs formulated using biodegradable polymers such as poly(lactic-*co*-glycolic acid) (PLGA). To boost immunological responses and raise the efficacy of immunotherapeutic treatments such as cancer vaccines the research group of Horvath and Basler [182], outlines several methods for refining PLGA NP formulations.

Furthermore, studies by Silva et al. [183] highlight the use of PLGA NPs for antigen delivery in immunotherapy and possible future directions and the difficulties associated with translating into clinical practice. Inorganic particles like gold and silica possess unique properties that enable targeted delivery. Antigen uptake can be improved by functionalizing these NPs to increase their selectivity towards APCs. Gold NPs (AuNPs) are used as carriers for antigen delivery in cancer immunotherapy, due to their unique properties of biocompatibility ease of functionalization, and capacity to target APCs. To increase immune response, promote antigen uptake, and ultimately improve the effectiveness of cancer vaccines, Huang et al. [184] highlight many approaches to alter AuNPs. Furthermore, they have also examined the challenges in the development of AuNP-based immuno-therapeutics [184, 185]. Silica NPs as carriers for targeted delivery of antigens also hold great potential for targeted delivery of antigens in immunotherapy. Silica NPs have unique surface chemistry, that can facilitate effective functionalization to increase the selectivity against APC [185, 186]. Since many APCs dwell in the skin, microneedle technology provides a less intrusive way to deliver antigens there. By improving antigen absorption by cutaneous DCs, this delivery technique can strengthen immune responses. Preclinical research on microneedle patches, which are intended as vaccinations against illnesses like the flu, has produced encouraging results. Nguyen [187] investigates the advancements in microneedle technology for transdermal drug delivery, focusing on its potential use in immunization. Microneedles can enhance immune responses by improving antigen delivery to skin-resident APCs. Moreover, it also emphasizes the potential application of microneedles targeting infectious diseases, exploring various microneedle designs, formulations, and both preclinical and clinical outcomes [187].

Nanotechnology may be used to administer pro-inflammatory cytokines (like IL-12 or IFN-γ) or block immunosuppressive ones (like TGF-β), changing the immune landscape toward an anti-tumor phenotype. This makes the cytokine milieu a crucial target. Nanomedicines based on their antigenic specificity, T cells destroy tumor cells and contribute to anti-tumor immune responses. Both CD4 and CD8 T cells can catalyze the breakdown of H_2_O_2_ inside tumors, respectively, moderating the role of T cells in controlling immunological responses. Furthermore, employing encapsulated small chemicals or genetic elements like siRNA, nanocarriers can target stromal cells, including TAMs, to polarize them from a pro-tumor M2 phenotype to an anti-tumor M1 phenotype. Additionally, by delivering PD-1/PD-L1 or CTLA-4 inhibitors, NPs can be engineered to interact with immune checkpoints, boosting anti-tumor immunity and reviving depleted T cells. These developments highlight how nanotechnology may completely alter the tumor immunological milieu, providing a variety of strategies to enhance treatment results [188].

The tumor immune microenvironment plays a crucial role in cancer progression and therapy, and nanotechnology offers promising strategies for its modulation. Key aspects targeted include the cytokine milieu, where NPs deliver pro-inflammatory cytokines or inhibit suppressive ones to enhance immune activation. Stromal cells, such as tumor-associated macrophages, are reprogrammed to reduce immunosuppression. Additionally, immune checkpoints, like PD-1/PD-L1, are targeted with nanocarriers to restore T-cell functionality, showcasing nanotechnology’s potential in advancing cancer immunotherapy [69, 188]

## Immunoinhibitor-NPs

Immunonanoparticles are created with improved targeting and efficacy, with an aim for reduced toxicity from dose and drug-resistant malignancy. Lipid-based NPs have been demonstrated in studies to improve the targeted transport of PD-1 inhibitors to tumor locations, hence lowering systemic exposure and raising local concentrations. This focused strategy reduces the negative consequences of systemic immunotherapy. Current research aims to understand the mechanisms underlying this resistance, which may help to create novel delivery methods or combination therapies [189]. The delivery of ICIs to tumor cells along with cytotoxic drugs was materialized using ADCs to lessen systemic toxicity and boost the local immune response. In preclinical models, this dual-action approach has demonstrated encouraging outcomes, especially for aggressive cancers. In combination therapy, ICI along with other forms of treatment radiation or chemotherapy has been more popular, as it can change the TME to make it more receptive to immune assault, thereby enhancing the delivery and efficacy of ICIs. ICI delivery can be improved by altering the TME. To increase the overall efficacy of ICIs, e.g., drugs that reduce immunosuppressive cells (such as Tregs) or stimulate immune cell infiltration can be used [190]. ICIs can be better stabilized and made more bioavailable by being encapsulated in NPs. In NPs, two different types of monoclonal antibodies (mAbs) against effector cells and tumor cells were combined. Anti-IgG (Fc specific) antibody (αFc) onto the NP surface (αFc-NP), and confirm that αFc-NP could conveniently and efficiently immobilize two types of mAbs through Fc-specific noncovalent interactions to form immunomodulating nano-adaptor (imNAs). Engineered formulation of imNA may bind to both cells and act as an “adaptor”, preserving the immunomodulatory qualities of the parent mAbs [191]. Combining siRNA therapy with chemotherapeutic drugs can overcome multidrug resistance and promote apoptosis. In a study, liposomes-protamine-hyaluronic acid (LPH)-NPs loaded with implantable blood clot scaffold containing both a vaccine and siRNA. LPH-siRNA that targets PD-L1 and TIM-3 can reduce immunosuppressive signals in mature DCs and prevent the DCs from expressing a regulatory program in the scaffold. The scaffold is intended to recruit immune cells, particularly DCs, to create a DC-rich environment and enhance the immune response [192]. ICIs such as PD-1 and CTLA-4 antibodies have demonstrated impressive effectiveness in a variety of malignancies. These inhibitors when encapsulated in NPs have shown better delivery at the tumor locations while reducing systemic adverse. To suppress PD-1 expression solid lipid NPs (SLNPs) were utilized to transport PD-1 siRNA to TAMs. The capacity of PD-1 siRNA-SLNPs to suppress PD-1 expression was verified in J774A.1 macrophage cell line in culture as well as in macrophages in B16-F10 tumors pre-established in mice. PD-1 siRNA-SLNPs significantly suppressed the growth of pre-existing B16-F10 tumors in mice when compared to siRNA-SLNPs generated using non-functional, negative control siRNA [193]. Riboxxim is an adjuvant for double-stranded RNA (dsRNA), incorporated into PLGA particles. Encapsulation of Riboxxim together with antigens potently activates murine and human DCs, resulting in increased tumor-specific CD8^+^ T cell responses that outperform those produced by traditional dsRNA analogs. In preclinical tumor models, the PLGA particle vaccination provides primary tumor growth retardation, metastasis prevention, and extended survival [194].

## Immunomodulators-NPs

Immunomodulation is the process of modifying the immune response to either stimulate or suppress certain immunological functions. Conventional immunotherapies, such as cytokines and mAbs, frequently have issues with adverse effects, specificity, and delivery [195]. On the other hand, NPs provide a flexible substrate that may be adapted for localized effects, controlled release, and targeted distribution. NPs can affect the immune system in several ways in which NPs function as immunomodulators [196].

Polymeric NP-based platforms can be designed to specifically modulate an immune system, e.g., it has been demonstrated that PLGA-NPs increase the delivery of adjuvants and antigens, resulting in better immune responses. Additionally, they may be made to release their payloads in a regulated way, which will allow for immune activation to continue over time [197]. TLR ligands and other immunomodulatory substances can be transported by PLGA particles to both the cytosol and endosome [198]. In a study, human peripheral blood monocytes were used to create iDCs, which were then treated with PLGA microparticles (MPs) or film. The maturation of the DCs was compared to either the negative control of untreated iDCs or the positive control of LPS treatment for DC maturation. In vitro study, the iDCs cultured with PLGA-MPs or PLGA film morphologically resembled that of LPS-matured DCs, and PLGA-MPs were associated with or may have internalized. Additionally, iDCs treated with biomaterials showed higher expression of costimulatory molecules and MHC class II than iDCs, but at a lower level than LPS-matured DCs. In vivo in mice, PLGA-MPs facilitated a moderate delayed-type hypersensitivity reaction due to DC maturation. Collectively, these findings imply that PLGA is a stimulant for DC maturation [198]. Kim et al. [170] demonstrated that adding TLR7/8 agonists to PLGA-NPs could dramatically boost co-stimulatory molecule production and antigen presentation in DCs. Studies conducted in vivo revealed that these NPs moved to the lymph nodes, where they activated and expanded DCs, resulting in increased CTL responses. This, in turn, improved the preventive and therapeutic efficacy of these NPs in tumor models of melanoma, bladder, and renal cell carcinoma [170]. PLGA was encapsulated with a STING agonist (cGAmicroparticle) and a small interfering RNA targeting SIRPα (si-SIRPα). Immunotherapeutic co-delivery method for the APCs’ phagocytosis checkpoint (signal regulatory protein α, SIRPα) silencer and stimulator of interferon genes (STING). In the ovalbumin-expressing B16-F10 (OVA-B16-F10) melanoma model, PLGA-NPs containing (si-SIRPα/cGAMP) reversed the immunosuppressive phenotype of APCs, promoting the activation of OVA-specific CD8^+^ T cells and generating comprehensive anti-tumor immune responses [199].

A nanoplatform was designed that allows for in situ tumor vaccination and the polarization of TAMs by employing a pH-sensitive triblock copolymer, that induced melanoma immunogenic cell death (ICD) through tertiary amines and thioethers. The nanocarrier itself targets mitochondria to control metabolism, causing endoplasmic reticulum stress and upregulating gasdermin D for pyroptosis, along with certain aspects of ferroptosis and apoptosis [200].

TAMs-targeted albumin NPs-based delivery system was designed for the co-delivery of photosensitizer IR820 and SHP2 inhibitor SHP099 to potentiate macrophage-mediated cancer immunotherapy. TAMs-targeted albumin NP remodeling resulted in the immunostimulatory TME by repolarizing TAMs to an M1 phenotype, restoring its phagocytic function and facilitating intratumoral CTLs infiltration, which significantly inhibited tumor growth [201].

Inorganic such as gold, silica, and iron oxide NPs display distinct optical and magnetic characteristics that can be used to modulate immune responses. Specifically, DCs may be activated by AuNPs via a variety of mechanisms, strengthening the immune system’s ability to combat malignancies [202]. Tricyclic ketolide, clarithromycin, and azithromycin are macrolide antibiotics that can aggregate in tumor-specific macrophages and induce cytotoxicity, which results in the killing of cancer cells. Dreaden et al. [203] used AuNPs in conjunction with these antibiotics. TAM cells were examined for preferential uptake/accumulation of macrolide-AuNPs using cardioid immersion darkfield scattering microscopy compared to non-malignant keratinocyte cells or squamous cell carcinoma. TAM cells (RAW 264.7) had significantly greater levels of macrolide-AuNPs absorption, however, AuNPs-activated macrophages might enhance TAMs’ natural cytotoxic reactions to the cancers they invade. Additionally, because of the size-dependent increased permeability and retention (EPR) effect, macrolide-AuNPs that specifically target TAMs can enhance anti-tumor response and provide better selective delivery [203]. Gold nanorods coated with BSA were created and utilized for photothermal ablation of breast cancer cells. Both photothermal ablation and photothermal conversion efficiency were high for the BSA-coated gold nanorods. The stimulatory effects of cell-cell interaction and soluble substances released by ablated tumor cells were confirmed by co-culturing the ablated tumor cells with iDCs utilizing both a diffusion model and a direct cell contacting paradigm. The breast tumor cells were efficiently ablated by the up-taken of BSA-coated gold nanorods after NIR laser irradiation [204]. In a study, M2-TAMs were reprogrammed toward M1-TAMs using hollow iron oxide (Fe_3_O_4_)-NPs to release proinflammatory cytokines and enlist T lymphocytes to destroy tumor cells. Hollow iron oxide (Fe_3_O_4_)-NPs were loaded with l-arginine (l-Arg) and sealed with poly(acrylic acid) (PAA). These NPs could release l-Arg in response to pH-responsive PAA and generate NO with the aid of inducible NO synthase (iNOS), which is overexpressed by M1-TAMs, as a result of further tumor removal for gas therapy. Synergistic tumor therapy could result from LPFe_3_O_4_ NPs’ ability to efficiently rewire M2 to M1 macrophages, activating T cells, releasing TNF-α, and generating large levels of NO, according to both in vitro and in vivo investigations [205].

Cell membrane alterations guarantee the structural stability of nanomaterials in challenging conditions and keep dangerous organic solvents out of the nanomaterials. Tumor immunotherapy is significantly mediated by tumor cell membranes. Tumor-associated antigens (TAAs) are frequently used in tumor immunotherapy to induce immune cell identification and start an immune response [206, 207]. Cancer cell membrane-coated-camouflaged MSNs loaded with dacarbazine combined with anti-PD-1 antibody provided enhanced antitumor effectiveness. In vivo results showed that combination therapy of chemotherapy and anti-PD-1 immunotherapy significantly suppresses the growth of melanoma and prolongs survival time, due to highly selective tumor killing, activation of tumor-specific T cells, and control of the immunosuppressive TME [208]. Table 1 describes some of the nanomaterials/NPs-based clinical trials.

**Table 1:** Summary of available clinical trials on nanomaterials-based tumor immunotherapy

**Nanomaterials**	**Cargo molecules**	**Indications**	**Clinical stage**	**Key findings**	**References**
Cyclodextrin polymer-based nanoparticle	Small interfering RNA targeting the M2 subunit of ribonucleotide reductase	Melanoma, gastrointestinal cancer, prostate cancer	Phase Ia/Ib	CALAA-01 pharmacokinetics revealed that peak concentration and exposure correlate with body weight across species.	[241]
Poly-*L*-lysine, double-stranded RNA complex with polyinosinic-polycytidylic acid	NY-ESO-1 antigen protein	Advanced or recurrent esophageal cancer	Phase I	Combination of CHP-NY-ESO-1 with poly-ICLC induced superior antigen-specific T-cell responses compared to monotherapy.	[242]
Poly-*L*-lysine, double-stranded RNA complex with polyinosinic-polycytidylic acid	NY-ESO-1 antigen protein + monomide	High-risk resected melanoma	Phase I/II	The CHP-NY-ESO-1 combination was well tolerated and effectively induced integrated CD4^+^ and CD8^+^ T-cell responses.	[243]
Poly-*L*-lysine pullulan (CHP) nanoparticle	NY-ESO-1 antigen protein	Esophageal cancer	Phase I (NCT01003868)	Tumor immunogenicity of the CHP-NY-ESO-1 vaccine was confirmed, showing promising therapeutic potential.	[244]
Nanoparticle albumin	HER2 protein 1–146	HER2-expressing solid cancer	Phase I	HER2-specific CD8^+^ T-cell responses were successfully detected in patients.	[245]
Nanoparticle albumin-bound (nab)-paclitaxel + atezolizumab (anti-PD-L1 antibody)	-	Unresectable locally advanced or metastatic triple-negative breast cancer	Phase III (NCT02425891)	Median progression-free survival was 7.5 months in PD-L1 positive patients treated with the nanoparticle-bound atezolizumab combination.	[246]
Poly (beta-amino ester) based nanomaterial	Plasmids encoding 19.4-1 BBZ CAR and a piggybac transposase	To be determined	Phase I (projected 2020–2021)	Efficient introduction of DNA plasmids leading to sustained disease remission with long-term therapeutic benefits.	[247]
IL-15 super-agonist complex	IL-15 super-agonist complex nanogel	Solid cancer and lymphomas	Phase I	The IL-15 super-agonist nanogel enabled high local cytokine concentrations while maintaining minimal systemic toxicity.	[248]

PD-L1: programmed death-ligand 1

*Note.* Adapted from “Nanomaterials in tumor immunotherapy: new strategies and challenges” by Zhu X, Li S. Mol Cancer. 2023;22:94 (https://molecular-cancer.biomedcentral.com/articles/10.1186/s12943-023-01797-9). CC BY.

## Nanoimmunotherapy for solid tumors

One of the most explored uses of nano-immunotherapy is cancer immunotherapy. Solid tumors produce lactic acid, which creates an acidic microenvironment that impairs T cells’ ability to respond to immunological stimuli [209]. An increase in lactate levels can lead to the inactivation and apoptosis of CD8^+^ T cells. Therefore, reversing lactic acid-induced cancers’ acidic microenvironment can be one of the ways to restore T cells’ immunological function [125]. Tumor acidity is influenced by the conversion of pyruvate to lactate, which is mostly carried out by lactate dehydrogenase A (LDHA). Restoring T cell anti-tumor function and reversing tumor acidity can be achieved by siRNA-mediated LDHA silencing. To silence LDHA in tumor cells, vascular CLAN NPs are used as a siRNA delivery system (VNPsi-LDHA). This therapy neutralized tumor acidity and dramatically decreased lactate generation, by which CD8^+^ T cell infiltration rose and their functionality was restored, leading to better anti-PD-1 immunotherapy [210]. ROS released by immunosuppressive cells in the TME may induce T cell apoptosis and functional inhibition resulting in sub-optimal T cell activity. To accomplish this, Lu et al. [210] created functionalized MnOx NPs (CD-MnOx@CM) that were camouflaged with tumor cell membranes (anti-CD3/CD28 mAbs, CD) to activate T cells and regulate TME. As expected, CDMnOx@CM efficiently activated CD8^+^ T cells and promoted T cell survival within the tumor by accelerating the breakdown of H_2_O_2_ into O_2_, which decreased ROS levels in the TME [210]. Tregs, a crucial subset of CD4^+^ T cells, maintain immunological homeostasis by suppressing immune responses [211]. Autoimmune disorders may result from aberrant Treg function and quantity [9]. Tregs play a role in tumor immunity by inhibiting anti-tumor immune responses [212]. By inhibiting effector T cells and causing monocytes to develop into the M2 subtype, which encourages tumor growth and metastasis, Tregs can cause tumor immune escape [213]. Furthermore, Treg-secreted TGF-β prevents immune cells from getting to the TME, which eventually results in TIME. Promoting an anti-tumor immune response now mostly involves inhibiting or removing Tregs.

Regulating redox metabolism of the TME, Tregs-mediated immunosuppression can be achieved. Fluorine-assembled NPs have been created to combat immunosuppression caused by Tregs [214]. By efficiently reducing hypoxia in the TME, the O_2_ included in perfluorocarbon-based fluorine-assembled NPs can prevent Treg invasion and proliferation. Conversely, when fluorine-assembled NPs are exposed to laser radiation, they lower GSH levels, which in turn lowers Foxp3 expression in Tregs and achieves the goal of reversing tumor immune suppression. Hybrid NPs functionalized with tLyp1 peptide for the delivery of imatinib. The tLyp1 peptide-conjugated hybrid NPs blocked the phosphorylation of STAT3 and STAT5, resulting in a decrease in Tregs within the tumors [215]. One potential target for triggering Treg expansion is the signaling pathway of mitogen-activated protein kinase (MEK)/extracellular signal-regulated kinase (ERK). For smart delivery of photosensitizers and immunomodulators. Demethylcantharidin (DMC)-conjugated b-cyclodextrin supramolecular photodynamic NPs. Supramolecular photodynamic NPs boosted CD8^+^ T cell infiltration and decreased Treg levels in TME, reversing TIME [216]. A combination of photodynamic therapy and imatinib action can lead to downregulation of Tregs in cancers. In this regard, encapsulating IR-780 and imatinib through layer-by-layer hybrid NPs displayed combined benefits of suppressing Tregs and anticancer activity [215].

Nano-curcumin can effectively decrease the upsurge of Tregs in tumors by inhibiting the MEK/ERK signaling pathway [217]. One important substance with immune-regulating qualities that comes from fruits and plants is ursolic acid (UA) [218]. Because UA liposomes prevent STAT5 phosphorylation and IL-10 release, they can decrease tumor-infiltrating Tregs [7]. Eliminating Tregs solely at the tumor site is more successful than doing so across the body because they are more directly linked to tumor immunosuppression in tumor tissue.

Although NP-mediated photothermal therapy (PTT) is frequently employed for local anticancer therapy, its capacity to eradicate Tregs has not been fully explored [219]. Therefore, PTT mediated by NPs might be a useful technique for boosting immunotherapy and locally eradicating Tregs. The combination anti-tumor activity of iron oxide NP-mediated PTT and CTLA-4115 was examined in mice breast carcinoma models. The findings demonstrated that iron oxide NP-mediated PTT can promote CTLA-4 suppression of tumor development and specifically kill Tregs at the tumor locations [148]. TAMs are supported by monocytes, which have been shown to actively contribute to tumor formation. TAMs, which include both native brain microglia and invading macrophages, can make up as much as 50% of the tumor mass cells in GBM [220].

Monocytes were delivered by conjugating to polymer NPs for an enhancement of PDT in GBM. Polymer NPs were conjugated with monocytes (murine monocytes derived from bone marrow and human monocytes derived from THP1) and assessed in GBM spheroids and an orthotopic model of the tumor. In the absence of light, the conjugated NPs could not exert any effect on monocyte viability, and following cell loading. Whereas, activated monocytes assimilated conjugated NPs in a higher percentage than that of naive monocytes. Cell-mediated administration of PDT was more effective in vitro than non-vehiculized CPNs [221].

MDSCs immature bone marrow cells that are crucial to TIME and have immunosuppressive properties also contribute to T cell immunological function inhibition [222]. Through nano-immunotherapy by reducing MDSC activity or preventing their recruitment can increase the anti-tumor efficacy [223]. The main way that nano-immunotherapy has increased anti-tumor efficacy is by reducing MDSC activity or preventing their recruitment. MDSCs express the scavenger receptor type B-1 (SCARB1), a high-affinity receptor for globular high-density lipoproteins (HDL). SCARB1 is specifically targeted by HDL NPs (HDL NPs). The findings indicated that HDL NPs decreased tumor development and MDSC activity considerably. Anti-tumor immunity is anticipated to be regulated by tadalafil through the inhibition of MDSC activity [224]. Co-delivering indocyanine green and tadalafil, often known as (FIT-NPs), delivered at tumor locations, released tadalafil can successfully suppress MDSC activity by strengthening PTT’s therapeutic effectiveness [225]. In tumor hypoxia, a hallmark of TME activates TIME by recruiting MDSCs [226]. Photosensitizer (IR780) and the mitochondrial respiratory inhibitor (metformin), CeO_2_ as the gatekeepers were coloaded to MSNs. In this method, O_2_ was produced in addition to the drug release. Crucially, Met’s involvement dramatically reduced mitochondrial respiration, acting as an O_2_ economizer. Consequently, by selectively reducing hypoxia at tumor sites, the populations and functions of tumor-infiltrated MDSCs were both significantly decreased, which helped to increase immune responses [227].

Tumor-derived G-CSF and GM-CSF are typically high in glycolysis along with the abundant LDHA, which further promotes the recruitment of MDSCs and immunosuppression. To achieve powerful anti-tumor immunity, an immunochemotherapy regimen based on a redox-responsive nanoassembly was developed (R-mPDV/PDV/DOX/siL). This redox-responsive nanoassembly is self-assembled by three glutathione (GSH)-responsive polymers that use 3, 3’-dithiodipropionic acid (DA) as a linkage to join hydrophilic segments and poly(δ-valerolactone) (PVL) as a hydrophobic segment. The core contains DOX and cationic PAMAM efficiently compresses LDHA siRNA (siL). The unique identification of integrin (αvβ3) by the c(RGDfk) (RGD) ligand strengthens the tumor-homing and cellular internalization. GSH-induced cleavage of DA causes R-mPDV/PDV/DOX/siL to disintegrate after escape from endosomes/lysosomes, resulting in burst drug release and highly effective LDHA silencing. The production of G-CSF and GM-CSF cytokines is suppressed, MDSC recruitment is limited, and anti-tumor immunity is strengthened when LDHA expression is decreased [228]. CAFs are one of the most abundant cells in the tumor matrix and play a crucial role in the development and metastasis of tumors, also the primary factor affecting TIME [229]. Cancer cells may proliferate as a result of the direct removal of CAFs, which would facilitate cancer invasion and metastasis. Thus, reprogramming active TAFs into a quiescent state for fine-tuning therapy is the best way to boost immune responses against tumors [230, 231]. Dendritic mesoporous silica was used as a nanocarrier to create tryptase-imprinted NPs (DMSN@MIPs), which neutralize TPS and prevent TAF activation. Moreover, combination therapy of DOX/LIP and DMSN@MIPs markedly altered TIME and raised the numbers of immune cells, including DCs, CD8^+^ T cells, and NK cells [232]. Zheng et al. [126], developed mPEG-modified PLGA NPs loaded with baicalin (Ba), for preventing TAF activation, promoting cytotoxic T cell infiltration, and activating the tumor immune milieu. An intravenous administration of Ba-loaded-PLGA-NPs along with Dox-NPs to the murine breast cancer model markedly increased the antitumor efficacy [233].

Cancer cells are forced to use TAFs as “energy factories”, providing the energy necessary for their quick development and multiplication. TAFs produce glycolytic metabolite lactate, which leads to impaired immune cell function, ultimately resulting in TIME. In this regard, concurrent suppression of glycolysis in TAFs and cancer cells may be essential for immune cell activity. Biomimetic NPs are developed by encapsulating paclitaxel into SLNPs that are modified with the membrane of cancer cells, aimed to target cancer cells by blocking the metabolic network between them, by blocking the glycolysis of cancer cells and the TAF decreased the lactate production in the TME and stimulated the immune responses, thereby improving the effectiveness of combined immunotherapy [234].

Additionally, preclinical studies show a 30–50% increase in CD8^+^ T-cell activation and 60% tumor regression in murine models. Clinical trials report a 6–12 month median survival extension, improved immune activation indices, and reduced immunosuppressive cell populations, underscoring its translational potential [126]. In addition, Alonso et al. [146] evaluated the safety and efficacy of OncoTherad^®^ (MRB-CFI-1) nanoimmunotherapy in treating non-muscle invasive bladder cancer (NMIBC) patients who did not respond to Bacillus Calmette-Guérin (BCG), while investigating its mechanisms of action within the bladder cancer microenvironment. Their studies show that it was a safe and effective therapeutic option for patients with BCG-unresponsive NMIBC, and also showed advantages in tumor relapse prevention processes [146].

Furthermore, quantitative data also highlights the efficacy of nanotechnology in cancer therapy. The pro-angiogenic effect of macrophages is one major reason for the failure of current anti-angiogenic therapies, as angiogenesis plays a key role in the progression and metastasis of melanoma. To inhibit melanoma, a nano-immunotherapy combining ferumoxytol and poly(I:C) [ferumoxytol/poly(I:C)] was developed to boost the anti-angiogenic activities of macrophages by the research group of Zheng et al. [126]. Their studies showed that ferumoxytol/poly(I:C) was a highly efficacious anti-tumor therapy and also limits angiogenesis [126]. In addition, Cano-Mejia et al. [235] developed a combination of nano-immunotherapy and PTT using CpG oligodeoxynucleotide-coated Prussian blue NPs (CpG-PB NPs). The PTT induced tumor cell death and facilitated the release of TAA. This approach enhanced immunogenicity in vitro and achieved remarkable results in a syngeneic neuroblastoma mouse model, with 70% of treated mice showing complete tumor regression and high survival rates at 60 days [235].

## Future perspectives

Despite advancements in ICI delivery, several challenges persist. Due to the heterogeneous nature of tumors, a treatment that works well for one type may not be effective for another. Additionally, the development of ICI resistance poses a significant challenge. Current research aims to understand the mechanism driving this resistance, which could facilitate the development of new delivery strategies or combination therapies. Furthermore, in clinical settings, novel delivery modalities’ safety profiles need to be carefully assessed. Even though preclinical research frequently shows promise, thorough clinical testing is necessary to convert these discoveries into useful treatments [236]. Ongoing research is crucial for overcoming existing challenges and improving ICI delivery systems. By optimizing their administration, the efficacy and broader application of these treatments can be enhanced. Even while NPs show promise as immunomodulators, there are still several issues that need to be resolved:

(i) **Safety and toxicity**: A detailed assessment is necessary to determine the long-term safety and biocompatibility of NPs. Although a large number of NPs exhibit minimal toxicity, it is unclear how they may ultimately affect immunological function. Following delivery, NPs interact with many innate and adaptive immune components and can affect immune systems in a variety of ways, from immunosuppression to immunomodulation, depending on their chemistry, composition, and design. The absorption, distribution, metabolism, and elimination (ADME) patterns of the NPs are subsequently impacted by their interactions with circulating macrophages and RES. Thus, the disposition pattern of NPs will differ greatly from that of any clinical subjects if specific pathogen-free (SFP) mice have immature immune or “neonate-like” immunity. The PK and pharmacodynamic (PD) activity of the drug-loaded NPs in the mouse model will always be significantly over-represented and this will result in a less realistic recapitulation when the NPs are injected into humans. Another important element influencing laboratory mice’s baseline immunity is their age; mice aged 6 weeks to 8 weeks are typically employed in vivo investigations. But age isn’t always a reliable indicator of immune system maturation. When laboratory-raised mice and feral mice were compared, it was evident that the latter had more naive lymphocytes [237] and a lower innate immune activation, which is similar to that of neonatal humans. Conversely, mice exposed to various microbiological challenges exhibit increased interferon and effector/memory cells, which are more like those found in adults. Therefore, research on the toxicity or effectiveness of NPs must be done on mice whose immunological system closely resembles that of human subjects. The immunological state of mice may be considerably impacted by SPF. Therefore, to facilitate the seamless transfer of NPs efficiency data from bench to bedside, a critical reassessment of animal models and alternative approaches should be taken into consideration. Currently, machine learning and systems biology have been proposed, to translate physiological and pathological correlations between species by incorporating a variety of molecular and phenotypic data from humans and animals, a modern method for humanizing computational models [238]. To improve the reliability of preclinical and clinical data matching, NP distribution in immunodeficient mice with physiologically similar human cytokine, chemokine, or ligand secretion should be studied.
(ii) **Regulatory obstacles**: Because comprehensive characterization and safety data are required, the approval procedure for medicines based on NPs might be difficult [239]. To guarantee the effectiveness and safety of these cutting-edge treatments, regulatory bodies demand extensive testing. Regulatory obstacles remain a significant challenge for NP-based therapies, as the approval process demands extensive characterization and robust safety data to address potential toxicity and long-term effects. Moreover, tumors can develop resistance to NPs through mechanisms like drug efflux or immune evasion, reducing treatment efficacy. Furthermore, off-target accumulation of NPs can cause unintended toxicity in healthy tissues, limiting their therapeutic potential. Addressing these challenges through targeted drug delivery, biomarker-driven approaches, and the development of novel nanomaterials is crucial for advancing the field and improving clinical outcomes. Additionally, unresolved challenges like scalability, cost-effectiveness, and the need for interdisciplinary collaboration emphasize the importance of continued innovation and policy reform to accelerate clinical translation and therapeutic impact [239].
(iii) **Scale-up production**: The mass production of nanomaterials for commercialization under good manufacturing practices (GMP) is another issue that is seriously impeding the progress of nanomedicine. The deployment of nanoproducts for medical purposes may be limited by several nanodrug delivery systems that are not well suited for large-scale production because of painstaking preparation procedures or the high cost of raw ingredients. Furthermore, a significant disparity exists between the development of nanomedicines in academic and industrial contexts. Micrograms or milligrams of product are often produced in academic laboratories, while grams or kilograms are required for clinical trials, pre-clinical screening, and clinical use. Small changes in the production process can drastically change the product’s properties and therapeutic effect, making it difficult to scale up any laboratory technique and difficult to achieve batch-to-batch repeatability. To guarantee successful nano-manufacturing, a strong quality control system must be followed [142].
(iv) **Customization of treatments:** Individual differences in immunological responses demand customized strategies. Creating NPs that are customized to meet the needs of individual patients will enhance treatment outcomes. The distribution of adjuvants and antigens to APCs is an emerging area of research. Potential avenues for future research may be towards personalized vaccinations with the use of proteomics and genomics developments through which it is possible to create vaccinations that are specifically matched to each recipient’s immunological profile, for increasing their effectiveness. Sensation-responsive materials can be included in smart delivery systems to enable the on-demand release of adjuvants and antigens in response to certain physiological signals. Stimuli-response NPs may be designed that release adjuvants and antigens in response to certain triggers in the TME, and are likely to play a significant role in future delivery systems. For instance, responsive NPs to enzyme activity, pH shifts, or hypoxia can enable the targeted release of therapeutic drugs, increasing their effectiveness and lowering their systemic toxicity. This approach may also improve the targeting of immune cells within the TME. Efforts need to be made towards combining adjuvant and antigen delivery with additional treatment approaches such as checkpoint inhibitors, CART-cell therapy, or oncolytic viruses for synergistic treatment. There is great potential in combination treatments that offer several ways to combat the tumor, which can strengthen the immune system as a whole. Subsequent research endeavors ought to delve into the synergistic effects and ideal combinations that optimize therapeutic efficacy. Approaches to be made towards modulation of the microenvironment, that can concentrate on altering the TME to foster an environment that is more conducive to immune activation. This may entail administering adjuvants and antigens in combination with substances that modify immunosuppressive elements, such as tumor-associated macrophages or Tregs. These methods have the potential to enhance the effectiveness of immunotherapy by altering the TME. Further, improved APC targeting is essential to create more effective ways to deliver adjuvants and antigens directly to APCs in the TME. Targeting techniques might involve the use of tailored NPs that can negotiate the intricate TME architecture or compounds that bind selectively to APC surface receptors. T-cell activation and antigen absorption can both be enhanced by this focused strategy. The development of more efficient delivery methods will be aided by ongoing research into the molecular mechanisms of immune regulation and antigen presentation. Further, to guarantee novel delivery systems’ efficacy and safety in clinical settings, regulatory frameworks need to be set up.

## Conclusions

Nano-immunotherapy is based on ICIs and is two fast-developing sectors that are being propelled by research into improving delivery techniques and overcoming obstacles. Enhancing the treatment effectiveness and expanding the range of tumors for which ICIs can be used. Future research must prioritize customized delivery approaches that are suited to the distinct features of each tumor to optimize the advantages of immunotherapies for patients. A flexible platform for directly modifying immune responses is provided by NPs. Novel approaches to treating cancer and autoimmune illnesses are made possible by their capacity to engage with immune cells, improve targeted delivery, and regulate immune activity. NP technology development must continue to solve safety, standardization, and regulatory issues. Vaccines and immunotherapeutics depend on antigens and adjuvants efficiently reaching APCs. Enhancements in antigen stability and bioavailability can be addressed by advances in NP technology and sophisticated delivery methods, which will ultimately result in more potent therapies. Furthermore, a potential area of medicine is the practical use of nano-immunotherapy, especially in the treatment of autoimmune diseases and cancer. Future tactics can improve the efficacy of immunotherapeutic treatments by utilizing combination medicines, stimuli-responsive systems, and tailored techniques. Transforming these discoveries into therapeutic practice will need interdisciplinary cooperation. The fields of artificial intelligence and nanotechnology have played a significant role in achieving precision medicine’s objective of customizing the optimal course of treatment for every cancer patient.

AI techniques, use pattern analysis and classification algorithms to increase the accuracy of diagnosis and treatment. Using AI to optimize material properties based on anticipated interactions with the target medication, biological fluids, immune system, vasculature, and cell membranes can impact therapeutic efficacy that benefits nanomedicine design. Computational techniques are crucial to the application of precision medicine. Numerous research has shown the efficacy of AI algorithms for precision medicine in patient classification, drug appropriateness screening, and nanomedicine property optimization. However, some obstacles need to be overcome before these algorithms may be used in therapeutic settings. The acquisition of sizable datasets for algorithm training is one of the most crucial challenges for attaining high accuracy in these computational techniques. Additionally, improved collaboration amongst specialists in computer science, medicine, and nanomaterials as well as the use of computing at every level of academic and commercial research would enhance their effectiveness and clinical applicability. New findings on NP uses and delivery systems are driving a revolution in immunotherapy. In the years to come, this combination of cutting-edge technologies and customized medicine has the potential to significantly improve patient outcomes and change treatment modalities.

## Abbreviations

aLNPs: activating lipid nanoparticles
APCs: antigen-presenting cells
AuNPs: gold nanoparticles
BSA: bovine serum albumin
CAFs: cancer-associated fibroblasts
CAR: chimeric antigen receptor
CAR-M: chimeric antigen receptor-macrophages
CRS: cytokine release system
CSNK2A1: casein kinase II
CTCs: circulating tumor cells
CTLA-4: cytotoxic T lymphocyte-associated antigen-4
CTLs: cytotoxic T lymphocytes
DCs: dendritic cells
ECM: extracellular matrix
ECs: endothelial cells
EPA: epacadostat
EPR: enhanced permeability and retention
GBM: glioblastoma multiforme
GM-CSF: granulocyte monocyte colony-stimulating factor
HDL: high-density lipoproteins
ICIs: immune checkpoint inhibitors
iDC: immature dendritic cell
IFN-γ: interferon-gamma
IL-2: interleukin-2
LDHA: lactate dehydrogenase A
LPS: lipopolysaccharide
mAbs: monoclonal antibodies
MDSCs: myeloid-derived suppressor cells
MMPs: matrix metalloproteinases
MPs: microparticles
MSNs: mesoporous silica nanoparticles
NIR: near-infrared radiation
NK: natural killer
NO: nitric oxide
NPs: nanoparticles
OmlA: outer membrane lipoprotein A
PD-1: programmed cell death-1
PD-L1: programmed death-ligand 1
PEG: polyethylene glycol
PKs: pharmacokinetics
PLGA: poly(lactic-*co*-glycolic acid)
pNIPAM: poly(*N*-isopropylacrylamide)
poly(I:C): polyinosinic-polycytidylic acid
PTT: photothermal therapy
RES: reticuloendothelial system
ROS: reactive oxygen species
scFv: single-chain variable fragment
siRNA: short interfering RNA
SLNPs: solid lipid nanoparticles
TAMs: tumor-associated macrophages
TANs: tumor-associated neutrophils
TGF-β: transforming growth factor-beta
TIME: tumor immunosuppressive microenvironment
TLR: Toll-like receptor
TME: tumor microenvironment
TNF-α: tumor necrosis factor-alpha
Tregs: regulatory T cells
US FDA: United States Food and Drug Administration
VEGF: vascular endothelial growth factor

## Declarations

### Copyright

© The Author(s) 2025.

## References

[1] Sun Y, Liu Y, Ma X, Hu H (2021). The Influence of Cell Cycle Regulation on Chemotherapy. Int J Mol Sci.

[2] Singh R, Malhotra A, Bansal R, Acharya PC, Kurosu M Chapter 15 - Synthetic cytotoxic drugs as cancer chemotherapeutic agents. Medicinal Chemistry of Chemotherapeutic Agents.

[3] Cella L, Monti S, Pacelli R, Palma G (2024). Modeling frameworks for radiation induced lymphopenia: A critical review. Radiother Oncol.

[4] Dunn SR, Bythell JC, Le Tissier MDA, Burnett WJ, Thomason JC (2002). Programmed cell death and cell necrosis activity during hyperthermic stress-induced bleaching of the symbiotic sea anemone *Aiptasia* sp. J Exp Mar Biol Ecol.

[5] Sobierajska K, Ciszewski WM, Sacewicz-Hofman I, Niewiarowska J (2020). Endothelial Cells in the Tumor Microenvironment. Adv Exp Med Biol.

[6] Wang B, Han Y, Zhang Y, Zhao Q, Wang H, Wei J (2023). Overcoming acquired resistance to cancer immune checkpoint therapy: potential strategies based on molecular mechanisms. Cell Biosci.

[7] Zhang N, Liu S, Shi S, Chen Y, Xu F, Wei X (2020). Solubilization and delivery of Ursolic-acid for modulating tumor microenvironment and regulatory T cell activities in cancer immunotherapy. J Control Release.

[8] Dagher C, Manning-Geist B, Ellenson LH, Weigelt B, Rios-Doria E, Barry D (2023). Molecular subtyping in endometrial cancer: A promising strategy to guide fertility preservation. Gynecol Oncol.

[9] Hossen MM, Ma Y, Yin Z, Xia Y, Du J, Huang JY (2023). Current understanding of CTLA-4: from mechanism to autoimmune diseases. Front Immunol.

[10] Wang F, Xia T, Li Z, Gao X, Fang X (2023). Current status of clinical trial research and application of immune checkpoint inhibitors for non-small cell lung cancer. Front Oncol.

[11] Ribas A, Wolchok JD (2013). Combining cancer immunotherapy and targeted therapy. Curr Opin Immunol.

[12] Li C, Jiang P, Wei S, Xu X, Wang J (2020). Regulatory T cells in tumor microenvironment: new mechanisms, potential therapeutic strategies and future prospects. Mol Cancer.

[13] Shafqat A, Omer MH, Ahmed EN, Mushtaq A, Ijaz E, Ahmed Z (2023). Reprogramming the immunosuppressive tumor microenvironment: exploiting angiogenesis and thrombosis to enhance immunotherapy. Front Immunol.

[14] Alatrash G, Jakher H, Stafford PD, Mittendorf EA (2013). Cancer immunotherapies, their safety and toxicity. Expert Opin Drug Saf.

[15] Hegde PS, Chen DS (2020). Top 10 Challenges in Cancer Immunotherapy. Immunity.

[16] Chiriva-Internati M, Bot A (2015). A new era in cancer immunotherapy: discovering novel targets and reprogramming the immune system. Int Rev Immunol.

[17] Zugazagoitia J, Guedes C, Ponce S, Ferrer I, Molina-Pinelo S, Paz-Ares L (2016). Current Challenges in Cancer Treatment. Clin Ther.

[18] Riley RS, June CH, Langer R, Mitchell MJ (2019). Delivery technologies for cancer immunotherapy. Nat Rev Drug Discov.

[19] Farooq MU, Lawrie CH, Deng NN (2024). Engineering nanoparticles for cancer immunotherapy: Current achievements, key considerations and future perspectives. Chem Eng J.

[20] Sadeghi F, Sanjari Moghaddam A, Soleyman-Jahi S, Rezaei N Hurdles in Cancer Immunotherapy. Cancer Immunology.

[21] Cai X, Jin M, Yao L, He B, Ahmed S, Safdar W (2023). Physicochemical properties, pharmacokinetics, toxicology and application of nanocarriers. J Mater Chem B.

[22] Kumar M, Hilles AR, Almurisi SH, Bhatia A, Mahmood S (2023). Micro and nano-carriers-based pulmonary drug delivery system: Their current updates, challenges, and limitations–A review. JCIS Open.

[23] Ding YN, Xue M, Tang QS, Wang LJ, Ding HY, Li H (2022). Immunotherapy-based novel nanoparticles in the treatment of gastrointestinal cancer: Trends and challenges. World J Gastroenterol.

[24] Chanmee T, Ontong P, Konno K, Itano N (2014). Tumor-associated macrophages as major players in the tumor microenvironment. Cancers (Basel).

[25] Benoit A, Vogin G, Duhem C, Berchem G, Janji B (2023). Lighting Up the Fire in the Microenvironment of Cold Tumors: A Major Challenge to Improve Cancer Immunotherapy. Cells.

[26] Zhang S, Li Z, Wang Q, Liu Q, Yuan W, Feng W (2022). An NIR-II Photothermally Triggered “Oxygen Bomb” for Hypoxic Tumor Programmed Cascade Therapy. Adv Mater.

[27] Chen F, Geng Z, Wang L, Zhou Y, Liu J (2022). Biomimetic Nanoparticles Enabled by Cascade Cell Membrane Coating for Direct Cross-Priming of T Cells. Small.

[28] Gascard P, Tlsty TD (2016). Carcinoma-associated fibroblasts: orchestrating the composition of malignancy. Genes Dev.

[29] Deyell M, Garris CS, Laughney AM (2021). Cancer metastasis as a non-healing wound. Br J Cancer.

[30] Nasrullah M, Meenakshi Sundaram DN, Claerhout J, Ha K, Demirkaya E, Uludag H (2023). Nanoparticles and cytokine response. Front Bioeng Biotechnol.

[31] Paunovska K, Loughrey D, Dahlman JE (2022). Drug delivery systems for RNA therapeutics. Nat Rev Genet.

[32] El-Andaloussi S, Lee Y, Lakhal-Littleton S, Li J, Seow Y, Gardiner C (2012). Exosome-mediated delivery of siRNA in vitro and in vivo. Nat Protoc.

[33] Xu L, Yeudall WA, Yang H (2017). Folic acid-decorated polyamidoamine dendrimer exhibits high tumor uptake and sustained highly localized retention in solid tumors: Its utility for local siRNA delivery. Acta Biomater.

[34] Li Y, Xu X (2020). Nanomedicine solutions to intricate physiological-pathological barriers and molecular mechanisms of tumor multidrug resistance. J Control Release.

[35] Cao J, Huang D, Peppas NA (2020). Advanced engineered nanoparticulate platforms to address key biological barriers for delivering chemotherapeutic agents to target sites. Adv Drug Deliv Rev.

[36] Dai L, Zhu W, Si C, Lei J (2018). “Nano-Ginseng” for Enhanced Cytotoxicity AGAINST Cancer Cells. Int J Mol Sci.

[37] Nienhaus K, Nienhaus GU (2023). Mechanistic Understanding of Protein Corona Formation around Nanoparticles: Old Puzzles and New Insights. Small.

[38] Mills JA, Liu F, Jarrett TR, Fletcher NL, Thurecht KJ (2022). Nanoparticle based medicines: approaches for evading and manipulating the mononuclear phagocyte system and potential for clinical translation. Biomater Sci.

[39] Lu J, Gao X, Wang S, He Y, Ma X, Zhang T (2023). Advanced strategies to evade the mononuclear phagocyte system clearance of nanomaterials. Exploration (Beijing).

[40] Durymanov MO, Rosenkranz AA, Sobolev AS (2015). Current Approaches for Improving Intratumoral Accumulation and Distribution of Nanomedicines. Theranostics.

[41] Huang W, Zhang L, Yang M, Wu X, Wang X, Huang W (2021). Cancer-associated fibroblasts promote the survival of irradiated nasopharyngeal carcinoma cells via the NF-κB pathway. J Exp Clin Cancer Res.

[42] Zalba S, Ten Hagen TLM, Burgui C, Garrido MJ (2022). Stealth nanoparticles in oncology: Facing the PEG dilemma. J Control Release.

[43] Jiang Z, Chu Y, Zhan C (2022). Protein corona: challenges and opportunities for targeted delivery of nanomedicines. Expert Opin Drug Deliv.

[44] Kaur M, Shivgotra R, Bhardwaj N, Saini S, Thakur S, Jain SK (2023). Nascent Nanoformulations as an Insight into the Limitations of the Conventional Systemic Antifungal Therapies. Curr Drug Targets.

[45] Vasan N, Baselga J, Hyman DM (2019). A view on drug resistance in cancer. Nature.

[46] Maeda H (2015). Toward a full understanding of the EPR effect in primary and metastatic tumors as well as issues related to its heterogeneity. Adv Drug Deliv Rev.

[47] Fam SY, Chee CF, Yong CY, Ho KL, Mariatulqabtiah AR, Tan WS (2020). Stealth Coating of Nanoparticles in Drug-Delivery Systems. Nanomaterials (Basel).

[48] Petersen GH, Alzghari SK, Chee W, Sankari SS, La-Beck NM (2016). Meta-analysis of clinical and preclinical studies comparing the anticancer efficacy of liposomal versus conventional non-liposomal doxorubicin. J Control Release.

[49] Danhier F (2016). To exploit the tumor microenvironment: Since the EPR effect fails in the clinic, what is the future of nanomedicine?. J Control Release.

[50] Biffi S, Voltan R, Bortot B, Zauli G, Secchiero P (2019). Actively targeted nanocarriers for drug delivery to cancer cells. Expert Opin Drug Deliv.

[51] Tylawsky DE, Kiguchi H, Vaynshteyn J, Gerwin J, Shah J, Islam T (2023). P-selectin-targeted nanocarriers induce active crossing of the blood-brain barrier via caveolin-1-dependent transcytosis. Nat Mater.

[52] Anarjan FS (2019). Active targeting drug delivery nanocarriers: Ligands. Nano Struct Nano Objects.

[53] Guo L, Yang J, Wang H, Yi Y (2023). Multistage Self-Assembled Nanomaterials for Cancer Immunotherapy. Molecules.

[54] Khongorzul P, Ling CJ, Khan FU, Ihsan AU, Zhang J (2020). Antibody-Drug Conjugates: A Comprehensive Review. Mol Cancer Res.

[55] Dumontet C, Demangel D, Galia P, Karlin L, Roche L, Fauvernier M (2023). Clinical characteristics and outcome of 318 families with familial monoclonal gammopathy: A multicenter Intergroupe Francophone du Myélome study. Am J Hematol.

[56] Joubert F, Munson MJ, Sabirsh A, England RM, Hemmerling M, Alexander C (2023). Precise and systematic end group chemistry modifications on PAMAM and poly(l-lysine) dendrimers to improve cytosolic delivery of mRNA. J Control Release.

[57] Samec T, Alatise KL, Boulos J, Gilmore S, Hazelton A, Coffin C (2022). Fusogenic peptide delivery of bioactive siRNAs targeting CSNK2A1 for treatment of ovarian cancer. Mol Ther Nucleic Acids.

[58] Zhang L, Zhu D, Dong X, Sun H, Song C, Wang C (2015). Folate-modified lipid-polymer hybrid nanoparticles for targeted paclitaxel delivery. Int J Nanomedicine.

[59] Qian Z, LaRochelle JR, Jiang B, Lian W, Hard RL, Selner NG (2014). Early endosomal escape of a cyclic cell-penetrating peptide allows effective cytosolic cargo delivery. Biochemistry.

[60] Appelbaum JS, LaRochelle JR, Smith BA, Balkin DM, Holub JM, Schepartz A (2012). Arginine topology controls escape of minimally cationic proteins from early endosomes to the cytoplasm. Chem Biol.

[61] Rydström A, Deshayes S, Konate K, Crombez L, Padari K, Boukhaddaoui H (2011). Direct translocation as major cellular uptake for CADY self-assembling peptide-based nanoparticles. PLoS One.

[62] Juliano RL (2016). The delivery of therapeutic oligonucleotides. Nucleic Acids Res.

[63] Singh AV, Bhardwaj P, Laux P, Pradeep P, Busse M, Luch A (2024). AI and ML-based risk assessment of chemicals: predicting carcinogenic risk from chemical-induced genomic instability. Front Toxicol.

[64] de Visser KE, Joyce JA (2023). The evolving tumor microenvironment: From cancer initiation to metastatic outgrowth. Cancer Cell.

[65] Deng G, Giralt S, Chung DJ, Landau H, Siman J, Li QS (2020). Reduction of Opioid Use by Acupuncture in Patients Undergoing Hematopoietic Stem Cell Transplantation: Secondary Analysis of a Randomized, Sham-Controlled Trial. Pain Med.

[66] Vignali DA, Collison LW, Workman CJ (2008). How regulatory T cells work. Nat Rev Immunol.

[67] Okeke EB, Uzonna JE (2019). The Pivotal Role of Regulatory T Cells in the Regulation of Innate Immune Cells. Front Immunol.

[68] Saleh R, Elkord E (2019). Treg-mediated acquired resistance to immune checkpoint inhibitors. Cancer Lett.

[69] Darya GH, Zare O, Karbalaei-Heidari HR, Zeinali S, Sheardown H, Rastegari B (2024). Enzyme-responsive mannose-grafted magnetic nanoparticles for breast and liver cancer therapy and tumor-associated macrophage immunomodulation. Expert Opin Drug Deliv.

[70] Zhu Y, An X, Zhang X, Qiao Y, Zheng T, Li X (2019). STING: a master regulator in the cancer-immunity cycle. Mol Cancer.

[71] Schmitt EG, Haribhai D, Williams JB, Aggarwal P, Jia S, Charbonnier LM (2012). IL-10 produced by induced regulatory T cells (iTregs) controls colitis and pathogenic ex-iTregs during immunotherapy. J Immunol.

[72] Coënon L, Geindreau M, Ghiringhelli F, Villalba M, Bruchard M (2024). Natural Killer cells at the frontline in the fight against cancer. Cell Death Dis.

[73] Ogura K, Sato-Matsushita M, Yamamoto S, Hori T, Sasahara M, Iwakura Y (2018). NK Cells Control Tumor-Promoting Function of Neutrophils in Mice. Cancer Immunol Res.

[74] Lin Y, Xu J, Lan H (2019). Tumor-associated macrophages in tumor metastasis: biological roles and clinical therapeutic applications. J Hematol Oncol.

[75] Basu B, Garala KK, Patel R, Dutta A, Ash D, Prajapati B (2025). Advanced Targeted Drug Delivery of Bioactive Nanomaterials in the Management of Cancer. Curr Med Chem.

[76] Chetty C, Lakka SS, Bhoopathi P, Rao JS (2010). MMP-2 alters VEGF expression via alphaVbeta3 integrin-mediated PI3K/AKT signaling in A549 lung cancer cells. Int J Cancer.

[77] Zhao Y, Shen M, Wu L, Yang H, Yao Y, Yang Q (2023). Stromal cells in the tumor microenvironment: accomplices of tumor progression?. Cell Death Dis.

[78] Hegde M, Bhat SM, Guruprasad KP, Moka R, Ramachandra L, Satyamoorthy K (2022). Human breast tumor derived endothelial cells exhibit distinct biological properties. Biol Cell.

[79] Hrabák P, Kalousová M, Krechler T, Zima T (2021). Pancreatic stellate cells - rising stars in pancreatic pathologies. Physiol Res.

[80] Doran AC, Yurdagul A Jr, Tabas I (2020). Efferocytosis in health and disease. Nat Rev Immunol.

[81] Boada-Romero E, Martinez J, Heckmann BL, Green DR (2020). The clearance of dead cells by efferocytosis. Nat Rev Mol Cell Biol.

[82] DeNardo DG, Ruffell B (2019). Macrophages as regulators of tumour immunity and immunotherapy. Nat Rev Immunol.

[83] Li Q, Liu H, Yin G, Xie Q (2024). Efferocytosis: Current status and future prospects in the treatment of autoimmune diseases. Heliyon.

[84] Kienle K, Lämmermann T (2016). Neutrophil swarming: an essential process of the neutrophil tissue response. Immunol Rev.

[85] Fridlender ZG, Sun J, Kim S, Kapoor V, Cheng G, Ling L (2009). Polarization of tumor-associated neutrophil phenotype by TGF-beta: “N1” versus “N2” TAN. Cancer Cell.

[86] Shaul ME, Fridlender ZG (2019). Tumour-associated neutrophils in patients with cancer. Nat Rev Clin Oncol.

[87] Ardi VC, Kupriyanova TA, Deryugina EI, Quigley JP (2007). Human neutrophils uniquely release TIMP-free MMP-9 to provide a potent catalytic stimulator of angiogenesis. Proc Natl Acad Sci U S A.

[88] Zhang J, Qiao X, Shi H, Han X, Liu W, Tian X (2016). Circulating tumor-associated neutrophils (cTAN) contribute to circulating tumor cell survival by suppressing peripheral leukocyte activation. Tumour Biol.

[89] Szczerba BM, Castro-Giner F, Vetter M, Krol I, Gkountela S, Landin J (2019). Neutrophils escort circulating tumour cells to enable cell cycle progression. Nature.

[90] Lambert AW, Pattabiraman DR, Weinberg RA (2017). Emerging Biological Principles of Metastasis. Cell.

[91] Ciernikova S, Sevcikova A, Stevurkova V, Mego M (2022). Tumor microbiome - an integral part of the tumor microenvironment. Front Oncol.

[92] Rossi T, Vergara D, Fanini F, Maffia M, Bravaccini S, Pirini F (2020). Microbiota-Derived Metabolites in Tumor Progression and Metastasis. Int J Mol Sci.

[93] Jin S, Leach JC, Ye K (2009). Nanoparticle-mediated gene delivery. Methods Mol Biol.

[94] Nejman D, Livyatan I, Fuks G, Gavert N, Zwang Y, Geller LT (2020). The human tumor microbiome is composed of tumor type-specific intracellular bacteria. Science.

[95] Yu T, Guo F, Yu Y, Sun T, Ma D, Han J (2017). Fusobacterium nucleatum Promotes Chemoresistance to Colorectal Cancer by Modulating Autophagy. Cell.

[96] Cohen AD, Garfall AL, Stadtmauer EA, Melenhorst JJ, Lacey SF, Lancaster E (2019). B cell maturation antigen-specific CAR T cells are clinically active in multiple myeloma. J Clin Invest.

[97] Geller LT, Barzily-Rokni M, Danino T, Jonas OH, Shental N, Nejman D (2017). Potential role of intratumor bacteria in mediating tumor resistance to the chemotherapeutic drug gemcitabine. Science.

[98] Vande Voorde J, Sabuncuoğlu S, Noppen S, Hofer A, Ranjbarian F, Fieuws S (2014). Nucleoside-catabolizing enzymes in mycoplasma-infected tumor cell cultures compromise the cytostatic activity of the anticancer drug gemcitabine. J Biol Chem.

[99] Huang Z, Callmann CE, Wang S, Vasher MK, Evangelopoulos M, Petrosko SH (2022). Rational Vaccinology: Harnessing Nanoscale Chemical Design for Cancer Immunotherapy. ACS Cent Sci.

[100] Wang M, Cao JX, Liu YS, Xu BL, Li D, Zhang XY (2015). Evaluation of tumour vaccine immunotherapy for the treatment of advanced non-small cell lung cancer: a systematic meta-analysis. BMJ Open.

[101] Maji M, Mazumder S, Bhattacharya S, Choudhury ST, Sabur A, Shadab M (2016). A Lipid Based Antigen Delivery System Efficiently Facilitates MHC Class-I Antigen Presentation in Dendritic Cells to Stimulate CD8(+) T Cells. Sci Rep.

[102] Filipić B, Pantelić I, Nikolić I, Majhen D, Stojić-Vukanić Z, Savić S (2023). Nanoparticle-Based Adjuvants and Delivery Systems for Modern Vaccines. Vaccines (Basel).

[103] Gu D, Ao X, Yang Y, Chen Z, Xu X (2018). Soluble immune checkpoints in cancer: production, function and biological significance. J Immunother Cancer.

[104] Marei HE, Hasan A, Pozzoli G, Cenciarelli C (2023). Cancer immunotherapy with immune checkpoint inhibitors (ICIs): potential, mechanisms of resistance, and strategies for reinvigorating T cell responsiveness when resistance is acquired. Cancer Cell Int.

[105] Zhang Y, Lin S, Wang XY, Zhu G (2019). Nanovaccines for cancer immunotherapy. Wiley Interdiscip Rev Nanomed Nanobiotechnol.

[106] Bhardwaj P, Bhatia E, Sharma S, Ahamad N, Banerjee R (2020). Advancements in prophylactic and therapeutic nanovaccines. Acta Biomater.

[107] Poudel K, Vithiananthan T, Kim JO, Tsao H (2025). Recent progress in cancer vaccines and nanovaccines. Biomaterials.

[108] Park JH, Geyer MB, Brentjens RJ (2016). CD19-targeted CAR T-cell therapeutics for hematologic malignancies: interpreting clinical outcomes to date. Blood.

[109] Zhang ZZ, Wang T, Wang XF, Zhang YQ, Song SX, Ma CQ (2022). Improving the ability of CAR-T cells to hit solid tumors: Challenges and strategies. Pharmacol Res.

[110] Murciano-Goroff YR, Warner AB, Wolchok JD (2020). The future of cancer immunotherapy: microenvironment-targeting combinations. Cell Res.

[111] Cappell KM, Kochenderfer JN (2023). Long-term outcomes following CAR T cell therapy: what we know so far. Nat Rev Clin Oncol.

[112] Boccalatte F, Mina R, Aroldi A, Leone S, Suryadevara CM, Placantonakis DG (2022). Advances and Hurdles in CAR T Cell Immune Therapy for Solid Tumors. Cancers (Basel).

[113] Roex G, Timmers M, Wouters K, Campillo-Davo D, Flumens D, Schroyens W (2020). Safety and clinical efficacy of BCMA CAR-T-cell therapy in multiple myeloma. J Hematol Oncol.

[114] Wang Z, Li W, Jiang Y, Tran TB, Cordova LE, Chung J (2023). Sphingomyelin-derived nanovesicles for the delivery of the IDO1 inhibitor epacadostat enhance metastatic and post-surgical melanoma immunotherapy. Nat Commun.

[115] Metzloff AE, Padilla MS, Gong N, Billingsley MM, Han X, Merolle M (2024). Antigen Presenting Cell Mimetic Lipid Nanoparticles for Rapid mRNA CAR T Cell Cancer Immunotherapy. Adv Mater.

[116] Maalej KM, Merhi M, Inchakalody VP, Mestiri S, Alam M, Maccalli C (2023). CAR-cell therapy in the era of solid tumor treatment: current challenges and emerging therapeutic advances. Mol Cancer.

[117] Klichinsky M, Ruella M, Shestova O, Lu XM, Best A, Zeeman M (2020). Human chimeric antigen receptor macrophages for cancer immunotherapy. Nat Biotechnol.

[118] Flores-Villanueva P, Sobhani N, Wang X, Li Y (2020). MR1-Restricted T Cells in Cancer Immunotherapy. Cancers (Basel).

[119] Boutilier AJ, Elsawa SF (2021). Macrophage Polarization States in the Tumor Microenvironment. Int J Mol Sci.

[120] Zhou J, Tang Z, Gao S, Li C, Feng Y, Zhou X (2020). Tumor-Associated Macrophages: Recent Insights and Therapies. Front Oncol.

[121] Tan Y, Wang M, Yang K, Chi T, Liao Z, Wei P (2021). PPAR-α Modulators as Current and Potential Cancer Treatments. Front Oncol.

[122] Sloas C, Gill S, Klichinsky M (2021). Engineered CAR-Macrophages as Adoptive Immunotherapies for Solid Tumors. Front Immunol.

[123] Liu M, Liu J, Liang Z, Dai K, Gan J, Wang Q (2022). CAR-Macrophages and CAR-T Cells Synergistically Kill Tumor Cells In Vitro. Cells.

[124] An M, Liu H (2017). Dissolving Microneedle Arrays for Transdermal Delivery of Amphiphilic Vaccines. Small.

[125] Chen Y, Yu Z, Tan X, Jiang H, Xu Z, Fang Y (2021). CAR-macrophage: A new immunotherapy candidate against solid tumors. Biomed Pharmacother.

[126] Zheng Y, Jiang B, Guo H, Zhang Z, Chen B, Zhang Z (2023). The combinational nano-immunotherapy of ferumoxytol and poly(I:C) inhibits melanoma via boosting anti-angiogenic immunity. Nanomedicine.

[127] Ye Z, Chen J, Zhao X, Li Y, Harmon J, Huang C (2022). In Vitro Engineering Chimeric Antigen Receptor Macrophages and T Cells by Lipid Nanoparticle-Mediated mRNA Delivery. ACS Biomater Sci Eng.

[128] Zhou Y, Bian P, Du H, Wang T, Li M, Hu H (2022). The Comparison of Inflammatory Cytokines (IL-6 and IL-18) and Immune Cells in Japanese Encephalitis Patients With Different Progression. Front Cell Infect Microbiol.

[129] Kang M, Lee SH, Kwon M, Byun J, Kim D, Kim C (2021). Nanocomplex-Mediated In Vivo Programming to Chimeric Antigen Receptor-M1 Macrophages for Cancer Therapy. Adv Mater.

[130] Shin MH, Kim J, Lim SA, Kim J, Kim SJ, Lee KM (2020). NK Cell-Based Immunotherapies in Cancer. Immune Netw.

[131] Iyoda T, Yamasaki S, Ueda S, Shimizu K, Fujii SI (2023). Natural Killer T and Natural Killer Cell-Based Immunotherapy Strategies Targeting Cancer. Biomolecules.

[132] Glasner A, Ghadially H, Gur C, Stanietsky N, Tsukerman P, Enk J (2012). Recognition and prevention of tumor metastasis by the NK receptor NKp46/NCR1. J Immunol.

[133] Wu SY, Fu T, Jiang YZ, Shao ZM (2020). Natural killer cells in cancer biology and therapy. Mol Cancer.

[134] Ramírez-Labrada A, Pesini C, Santiago L, Hidalgo S, Calvo-Pérez A, Oñate C (2022). All About (NK Cell-Mediated) Death in Two Acts and an Unexpected Encore: Initiation, Execution and Activation of Adaptive Immunity. Front Immunol.

[135] Smyth MJ, Crowe NY, Godfrey DI (2001). NK cells and NKT cells collaborate in host protection from methylcholanthrene-induced fibrosarcoma. Int Immunol.

[136] Oberoi P, Kamenjarin K, Ossa JFV, Uherek B, Bönig H, Wels WS (2020). Directed Differentiation of Mobilized Hematopoietic Stem and Progenitor Cells into Functional NK cells with Enhanced Antitumor Activity. Cells.

[137] Lu H, Zhao X, Li Z, Hu Y, Wang H (2021). From CAR-T Cells to CAR-NK Cells: A Developing Immunotherapy Method for Hematological Malignancies. Front Oncol.

[138] Gong Y, Klein Wolterink RGJ, Wang J, Bos GMJ, Germeraad WTV (2021). Chimeric antigen receptor natural killer (CAR-NK) cell design and engineering for cancer therapy. J Hematol Oncol.

[139] Shin S, Lee P, Han J, Kim SN, Lim J, Park DH (2023). Nanoparticle-Based Chimeric Antigen Receptor Therapy for Cancer Immunotherapy. Tissue Eng Regen Med.

[140] McKinlay CJ, Vargas JR, Blake TR, Hardy JW, Kanada M, Contag CH (2017). Charge-altering releasable transporters (CARTs) for the delivery and release of mRNA in living animals. Proc Natl Acad Sci U S A.

[141] Kim KS, Han JH, Park JH, Kim HK, Choi SH, Kim GR (2019). Multifunctional nanoparticles for genetic engineering and bioimaging of natural killer (NK) cell therapeutics. Biomaterials.

[142] Dilnawaz F, Acharya S, Sahoo SK (2018). Recent trends of nanomedicinal approaches in clinics. Int J Pharm.

[143] Dilnawaz F, Singh A, Mohanty C, Sahoo SK (2010). Dual drug loaded superparamagnetic iron oxide nanoparticles for targeted cancer therapy. Biomaterials.

[144] Dilnawaz F, Singh A, Mewar S, Sharma U, Jagannathan NR, Sahoo SK (2012). The transport of non-surfactant based paclitaxel loaded magnetic nanoparticles across the blood brain barrier in a rat model. Biomaterials.

[145] Dilnawaz F, Sahoo SK (2013). Enhanced accumulation of curcumin and temozolomide loaded magnetic nanoparticles executes profound cytotoxic effect in glioblastoma spheroid model. Eur J Pharm Biopharm.

[146] Alonso JCC, de Souza BR, Reis IB, de Arruda Camargo GC, de Oliveira G, de Barros Frazão Salmazo MI (2023). OncoTherad^® ^(MRB-CFI-1) Nanoimmunotherapy: A Promising Strategy to Treat Bacillus Calmette-Guérin-Unresponsive Non-Muscle-Invasive Bladder Cancer: Crosstalk among T-Cell CX3CR1, Immune Checkpoints, and the Toll-Like Receptor 4 Signaling Pathway. Int J Mol Sci.

[147] He S, Zhang L, Bai S, Yang H, Cui Z, Zhang X (2021). Advances of molecularly imprinted polymers (MIP) and the application in drug delivery. Eur Polym J.

[148] Chen YX, Wei CX, Lyu YQ, Chen HZ, Jiang G, Gao XL (2020). Biomimetic drug-delivery systems for the management of brain diseases. Biomater Sci.

[149] Chen Z, Hu Q, Gu Z (2018). Leveraging Engineering of Cells for Drug Delivery. Acc Chem Res.

[150] Kokate R (2017). A systematic overview of cancer immunotherapy: an emerging therapy. Pharm. Pharmacol Int J.

[151] Wu YH, Chen RJ, Chiu HW, Yang LX, Wang YL, Chen YY (2023). Nanoparticles augment the therapeutic window of RT and immunotherapy for treating cancers: pivotal role of autophagy. Theranostics.

[152] Zupančič E, Curato C, Paisana M, Rodrigues C, Porat Z, Viana AS (2017). Rational design of nanoparticles towards targeting antigen-presenting cells and improved T cell priming. J Control Release.

[153] (2024). Amulya Jindal, Mainuddin, Anoop Kumar, Ratneshwar Kumar Ratnesh. Nanotechnology Driven Lipid and Metalloid Based Formulations Targeting Blood–Brain Barrier (3B) for Brain Tumor. Indian J Microbiol.

[154] Yang M, Li J, Gu P, Fan X (2020). The application of nanoparticles in cancer immunotherapy: Targeting tumor microenvironment. Bioact Mater.

[155] Singh AV, Gemmati D, Kanase A, Pandey I, Misra V, Kishore V (2018). Nanobiomaterials for vascular biology and wound management: A review. Veins Lymphatics.

[156] Soriano Pérez ML, Funes JA, Flores Bracamonte C, Ibarra LE, Forrellad MA, Taboga O (2023). Development and biological evaluation of pNIPAM-based nanogels as vaccine carriers. Int J Pharm.

[157] Xiang M, Yang C, Zhang L, Wang S, Ren Y, Gou M (2024). Dissolving microneedles for transdermal drug delivery in cancer immunotherapy. J Mater Chem B.

[158] Li N, Peng LH, Chen X, Nakagawa S, Gao JQ (2011). Transcutaneous vaccines: novel advances in technology and delivery for overcoming the barriers. Vaccine.

[159] He Y, Hong C, Li J, Howard MT, Li Y, Turvey ME (2018). Synthetic Charge-Invertible Polymer for Rapid and Complete Implantation of Layer-by-Layer Microneedle Drug Films for Enhanced Transdermal Vaccination. ACS Nano.

[160] Lee SJ, Lee HS, Hwang YH, Kim JJ, Kang KY, Kim SJ (2019). Enhanced anti-tumor immunotherapy by dissolving microneedle patch loaded ovalbumin. PLoS One.

[161] Zhao JH, Zhang QB, Liu B, Piao XH, Yan YL, Hu XG (2017). Enhanced immunization via dissolving microneedle array-based delivery system incorporating subunit vaccine and saponin adjuvant. Int J Nanomedicine.

[162] Duong HTT, Yin Y, Thambi T, Kim BS, Jeong JH, Lee DS (2020). Highly potent intradermal vaccination by an array of dissolving microneedle polypeptide cocktails for cancer immunotherapy. J Mater Chem B.

[163] Wang C, Ye Y, Hochu GM, Sadeghifar H, Gu Z (2016). Enhanced Cancer Immunotherapy by Microneedle Patch-Assisted Delivery of Anti-PD1 Antibody. Nano Lett.

[164] Ye Y, Wang C, Zhang X, Hu Q, Zhang Y, Liu Q (2017). A melanin-mediated cancer immunotherapy patch. Sci Immunol.

[165] Vora LK, Moffatt K, Tekko IA, Paredes AJ, Volpe-Zanutto F, Mishra D (2021). Microneedle array systems for long-acting drug delivery. Eur J Pharm Biopharm.

[166] Lan X, Zhu W, Huang X, Yu Y, Xiao H, Jin L (2020). Microneedles loaded with anti-PD-1-cisplatin nanoparticles for synergistic cancer immuno-chemotherapy. Nanoscale.

[167] Chen SX, Ma M, Xue F, Shen S, Chen Q, Kuang Y (2020). Construction of microneedle-assisted co-delivery platform and its combining photodynamic/immunotherapy. J Control Release.

[168] Cole G, Ali AA, McErlean E, Mulholland EJ, Short A, McCrudden CM (2019). DNA vaccination via RALA nanoparticles in a microneedle delivery system induces a potent immune response against the endogenous prostate cancer stem cell antigen. Acta Biomater.

[169] Ali AA, McCrudden CM, McCaffrey J, McBride JW, Cole G, Dunne NJ (2017). DNA vaccination for cervical cancer; a novel technology platform of RALA mediated gene delivery via polymeric microneedles. Nanomedicine.

[170] Kim NW, Kim SY, Lee JE, Yin Y, Lee JH, Lim SY (2018). Enhanced Cancer Vaccination by In Situ Nanomicelle-Generating Dissolving Microneedles. ACS Nano.

[171] Li C, Wang J, Wang Y, Gao H, Wei G, Huang Y (2019). Recent progress in drug delivery. Acta Pharm Sin B.

[172] Gomes AC, Mohsen M, Bachmann MF (2017). Harnessing Nanoparticles for Immunomodulation and Vaccines. Vaccines (Basel).

[173] Schijns VEJC, O’Hagan DT Immunopotentiators in modern vaccines.

[174] Lindblad EB, Duroux L, Schijns VEJC, O’Hagan DT Mineral adjuvants. Immunopotentiators in Modern Vaccines (Second Edition).

[175] Wang Y, Yao C, Ding L, Li C, Wang J, Wu M (2017). Enhancement of the Immune Function by Titanium Dioxide Nanorods and Their Application in Cancer Immunotherapy. J Biomed Nanotechnol.

[176] Hem SL, Hogenesch H (2007). Relationship between physical and chemical properties of aluminum-containing adjuvants and immunopotentiation. Expert Rev Vaccines.

[177] Hess KL, Medintz IL, Jewell CM (2019). Designing inorganic nanomaterials for vaccines and immunotherapies. Nano Today.

[178] Tan K, Li R, Huang X, Liu Q (2018). Outer Membrane Vesicles: Current Status and Future Direction of These Novel Vaccine Adjuvants. Front Microbiol.

[179] Amini Y, Moradi B, Fasihi-Ramandi M (2017). Aluminum hydroxide nanoparticles show strong activity to stimulate Th-1 immune response against tuberculosis. Artif Cells Nanomed Biotechnol.

[180] Song C, Li F, Wang S, Wang J, Wei W, Ma G (2020). Recent advances in particulate adjuvants for cancer vaccination. Adv Ther.

[181] Alfagih IM, Aldosari B, AlQuadeib B, Almurshedi A, Alfagih MM (2020). Nanoparticles as Adjuvants and Nanodelivery Systems for mRNA-Based Vaccines. Pharmaceutics.

[182] Horvath D, Basler M (2023). PLGA Particles in Immunotherapy. Pharmaceutics.

[183] Silva AL, Soema PC, Slütter B, Ossendorp F, Jiskoot W (2016). PLGA particulate delivery systems for subunit vaccines: Linking particle properties to immunogenicity. Hum Vaccin Immunother.

[184] Huang H, Liu R, Yang J, Dai J, Fan S, Pi J (2023). Gold Nanoparticles: Construction for Drug Delivery and Application in Cancer Immunotherapy. Pharmaceutics.

[185] Heidegger S, Gössl D, Schmidt A, Niedermayer S, Argyo C, Endres S (2016). Immune response to functionalized mesoporous silica nanoparticles for targeted drug delivery. Nanoscale.

[186] Huang W, Pan H, Hu Z, Wang M, Wu L, Zhang F (2023). A functional bimodal mesoporous silica nanoparticle with redox/cellulase dual-responsive gatekeepers for controlled release of fungicide. Sci Rep.

[187] Nguyen HX (2023). Microneedles: The Future of Drug Delivery.

[188] Chandrasekar V, Panicker AJ, Dey AK, Mohammad S, Chakraborty A, Samal SK (2024). Integrated approaches for immunotoxicity risk assessment: challenges and future directions. Dis Toxicol.

[189] Dai W, Wang X, Song G, Liu T, He B, Zhang H (2017). Combination antitumor therapy with targeted dual-nanomedicines. Adv Drug Deliv Rev.

[190] Lee L, Gupta M, Sahasranaman S (2016). Immune Checkpoint inhibitors: An introduction to the next-generation cancer immunotherapy. J Clin Pharmacol.

[191] Lakshmanan VK, Jindal S, Packirisamy G, Ojha S, Lian S, Kaushik A (2021). Nanomedicine-based cancer immunotherapy: recent trends and future perspectives. Cancer Gene Ther.

[192] Chen R, Li Y, Zhuang Y, Zhang Y, Wu H, Lin T (2023). Immune evaluation of granulocyte-macrophage colony stimulating factor loaded hierarchically 3D nanofiber scaffolds in a humanized mice model. Front Bioeng Biotechnol.

[193] Hanafy MS, Hufnagel S, Trementozzi AN, Sakran W, Stachowiak JC, Koleng JJ (2021). PD-1 siRNA-Encapsulated Solid Lipid Nanoparticles Downregulate PD-1 Expression by Macrophages and Inhibit Tumor Growth: PD-1 siRNA-Encapsulated Solid Lipid Nanoparticles. AAPS PharmSciTech.

[194] Koerner J, Horvath D, Herrmann VL, MacKerracher A, Gander B, Yagita H (2021). PLGA-particle vaccine carrying TLR3/RIG-I ligand Riboxxim synergizes with immune checkpoint blockade for effective anti-cancer immunotherapy. Nat Commun.

[195] Wei Z, Yi Y, Luo Z, Gong X, Jiang Y, Hou D (2022). Selenopeptide Nanomedicine Activates Natural Killer Cells for Enhanced Tumor Chemoimmunotherapy. Adv Mater.

[196] Liu J, Liu Z, Pang Y, Zhou H (2022). The interaction between nanoparticles and immune system: application in the treatment of inflammatory diseases. J Nanobiotechnology.

[197] Lee EY, Srinivasan Y, de Anda J, Nicastro LK, Tükel Ç, Wong GCL (2020). Functional Reciprocity of Amyloids and Antimicrobial Peptides: Rethinking the Role of Supramolecular Assembly in Host Defense, Immune Activation, and Inflammation. Front Immunol.

[198] Yoshida M, Babensee JE (2004). Poly(lactic-co-glycolic acid) enhances maturation of human monocyte-derived dendritic cells. J Biomed Mater Res A.

[199] Lu ZD, Chen YF, Shen S, Xu CF, Wang J (2021). Co-delivery of Phagocytosis Checkpoint Silencer and Stimulator of Interferon Genes Agonist for Synergetic Cancer Immunotherapy. ACS Appl Mater Interfaces.

[200] Guo Y, Li Y, Zhang M, Ma R, Wang Y, Weng X (2024). Polymeric nanocarrier via metabolism regulation mediates immunogenic cell death with spatiotemporal orchestration for cancer immunotherapy. Nat Commun.

[201] Cheng L, Zhang P, Liu Y, Liu Z, Tang J, Xu L (2023). Multifunctional hybrid exosomes enhanced cancer chemo-immunotherapy by activating the STING pathway. Biomaterials.

[202] Yang Y, Huang J, Liu M, Qiu Y, Chen Q, Zhao T (2023). Emerging Sonodynamic Therapy-Based Nanomedicines for Cancer Immunotherapy. Adv Sci (Weinh).

[203] Dreaden EC, Alkilany AM, Huang X, Murphy CJ, El-Sayed MA (2012). The golden age: gold nanoparticles for biomedicine. Chem Soc Rev.

[204] Wang X, Li J, Kawazoe N, Chen G (2018). Photothermal Ablation of Cancer Cells by Albumin-Modified Gold Nanorods and Activation of Dendritic Cells. Materials (Basel).

[205] Wu X, Cheng Y, Zheng R, Xu K, Yan J, Song P (2021). Immunomodulation of Tumor Microenvironment by Arginine-Loaded Iron Oxide Nanoparticles for Gaseous Immunotherapy. ACS Appl Mater Interfaces.

[206] Yan T, Zhu L, Chen J (2023). Current advances and challenges in CAR T-Cell therapy for solid tumors: tumor-associated antigens and the tumor microenvironment. Exp Hematol Oncol.

[207] de Sousa E, Lérias JR, Beltran A, Paraschoudi G, Condeço C, Kamiki J (2021). Targeting Neoepitopes to Treat Solid Malignancies: Immunosurgery. Front Immunol.

[208] Zhu L, Liu J, Zhou G, Liu TM, Dai Y, Nie G (2021). Remodeling of Tumor Microenvironment by Tumor-Targeting Nanozymes Enhances Immune Activation of CAR T Cells for Combination Therapy. Small.

[209] Gao Y, Zhou H, Liu G, Wu J, Yuan Y, Shang A (2022). Tumor Microenvironment: Lactic Acid Promotes Tumor Development. J Immunol Res.

[210] Lu Y, Li L, Du J, Chen J, Xu X, Yang X (2021). Immunotherapy for Tumor Metastasis by Artificial Antigen-Presenting Cells via Targeted Microenvironment Regulation and T-Cell Activation. ACS Appl Mater Interfaces.

[211] Zhang W, Liu X, Zhu Y, Liu X, Gu Y, Dai X (2021). Transcriptional and posttranslational regulation of Th17/Treg balance in health and disease. Eur J Immunol.

[212] Huppert LA, Green MD, Kim L, Chow C, Leyfman Y, Daud AI (2022). Tissue-specific Tregs in cancer metastasis: opportunities for precision immunotherapy. Cell Mol Immunol.

[213] Liu C, Chikina M, Deshpande R, Menk AV, Wang T, Tabib T (2019). Treg Cells Promote the SREBP1-Dependent Metabolic Fitness of Tumor-Promoting Macrophages via Repression of CD8^+^ T Cell-Derived Interferon-γ. Immunity.

[214] Li Z, Deng Y, Sun H, Tan C, Li H, Mo F (2023). Redox modulation with a perfluorocarbon nanoparticle to reverse Treg-mediated immunosuppression and enhance anti-tumor immunity. J Control Release.

[215] Ou W, Jiang L, Thapa RK, Soe ZC, Poudel K, Chang JH (2018). Combination of NIR therapy and regulatory T cell modulation using layer-by-layer hybrid nanoparticles for effective cancer photoimmunotherapy. Theranostics.

[216] Chen Y, Zhou Q, Jia Z, Cheng N, Zhang S, Chen W (2024). Enhancing cancer immunotherapy: Nanotechnology-mediated immunotherapy overcoming immunosuppression. Acta Pharm Sin B.

[217] Hossain DM, Panda AK, Chakrabarty S, Bhattacharjee P, Kajal K, Mohanty S (2015). MEK inhibition prevents tumour-shed transforming growth factor-β-induced T-regulatory cell augmentation in tumour milieu. Immunology.

[218] Chauhan A, Pathak VM, Yadav M, Chauhan R, Babu N, Chowdhary M (2024). Role of ursolic acid in preventing gastrointestinal cancer: recent trends and future perspectives. Front Pharmacol.

[219] Kumar AVP, Dubey SK, Tiwari S, Puri A, Hejmady S, Gorain B (2021). Recent advances in nanoparticles mediated photothermal therapy induced tumor regression. Int J Pharm.

[220] Caverzán MD, Beaugé L, Oliveda PM, Cesca González B, Bühler EM, Ibarra LE (2023). Exploring Monocytes-Macrophages in Immune Microenvironment of Glioblastoma for the Design of Novel Therapeutic Strategies. Brain Sci.

[221] Ibarra LE, Beaugé L, Arias-Ramos N, Rivarola VA, Chesta CA, López-Larrubia P (2020). Trojan horse monocyte-mediated delivery of conjugated polymer nanoparticles for improved photodynamic therapy of glioblastoma. Nanomedicine (Lond).

[222] Veglia F, Sanseviero E, Gabrilovich DI (2021). Myeloid-derived suppressor cells in the era of increasing myeloid cell diversity. Nat Rev Immunol.

[223] Xu C, Amna N, Shi Y, Sun R, Weng C, Chen J (2024). Drug-Loaded Mesoporous Silica Nanoparticles Enhance Antitumor Immunotherapy by Regulating MDSCs. Molecules.

[224] Ghosh S, Johanns TM, Chheda MG, Liu E, Butt O, Abraham C (2023). A pilot phase Ib study to evaluate tadalafil to overcome immunosuppression during chemoradiotherapy for IDH-wild-type glioblastoma. Neurooncol Adv.

[225] Zhang T, Xiong H, Ma X, Gao Y, Xue P, Kang Y (2021). Supramolecular Tadalafil Nanovaccine for Cancer Immunotherapy by Alleviating Myeloid-Derived Suppressor Cells and Heightening Immunogenicity. Small Methods.

[226] Wang B, Zhao Q, Zhang Y, Liu Z, Zheng Z, Liu S (2021). Targeting hypoxia in the tumor microenvironment: a potential strategy to improve cancer immunotherapy. J Exp Clin Cancer Res.

[227] Zhou S, Li D, Lee C, Xie J (2020). Nanoparticle Phototherapy in the Era of Cancer Immunotherapy. Trends Chem.

[228] Xia C, Li M, Ran G, Wang X, Lu Z, Li T (2021). Redox-responsive nanoassembly restrained myeloid-derived suppressor cells recruitment through autophagy-involved lactate dehydrogenase A silencing for enhanced cancer immunochemotherapy. J Control Release.

[229] Knipper K, Lyu SI, Quaas A, Bruns CJ, Schmidt T (2023). Cancer-Associated Fibroblast Heterogeneity and Its Influence on the Extracellular Matrix and the Tumor Microenvironment. Int J Mol Sci.

[230] Ai W, Liu T, Lv C, Feng X, Wang Q (2023). Modulation of cancer-associated fibroblasts by nanodelivery system to enhance efficacy of tumor therapy. Nanomedicine (Lond).

[231] Fei B, Mo Z, Yang J, Wang Z, Li S (2023). Nanodrugs Reprogram Cancer-Associated Fibroblasts and Normalize Tumor Vasculatures for Sequentially Enhancing Photodynamic Therapy of Hepatocellular Carcinoma. Int J Nanomedicine.

[232] He Y, Hong C, Huang S, Kaskow JA, Covarrubias G, Pires IS (2023). STING Protein-Based In Situ Vaccine Synergizes CD4^+^ T, CD8^+^ T, and NK Cells for Tumor Eradication. Adv Healthc Mater.

[233] Li Y, Chen Z, Gu L, Duan Z, Pan D, Xu Z (2022). Anticancer nanomedicines harnessing tumor microenvironmental components. Expert Opin Drug Deliv.

[234] Zang S, Huang K, Li J, Ren K, Li T, He X (2022). Metabolic reprogramming by dual-targeting biomimetic nanoparticles for enhanced tumor chemo-immunotherapy. Acta Biomater.

[235] Cano-Mejia J, Shukla A, Ledezma DK, Palmer E, Villagra A, Fernandes R (2020). CpG-coated prussian blue nanoparticles-based photothermal therapy combined with anti-CTLA-4 immune checkpoint blockade triggers a robust abscopal effect against neuroblastoma. Transl Oncol.

[236] Singh D, Dilnawaz F, Sahoo SK (2020). Challenges of moving theranostic nanomedicine into the clinic. Nanomedicine (Lond).

[237] Reese TA, Bi K, Kambal A, Filali-Mouhim A, Beura LK, Bürger MC (2016). Sequential Infection with Common Pathogens Promotes Human-like Immune Gene Expression and Altered Vaccine Response. Cell Host Microbe.

[238] Brubaker DK, Lauffenburger DA (2020). Translating preclinical models to humans. Science.

[239] Singh AV, Bhardwaj P, Upadhyay AK, Pagani A, Upadhyay J, Bhadra J (2024). Navigating regulatory challenges in molecularly tailored nanomedicine. Explor BioMat-X.

[240] Zhu X, Li S (2023). Nanomaterials in tumor immunotherapy: new strategies and challenges. Mol Cancer.

[241] Zuckerman JE, Gritli I, Tolcher A, Heidel JD, Lim D, Morgan R (2014). Correlating animal and human phase Ia/Ib clinical data with CALAA-01, a targeted, polymer-based nanoparticle containing siRNA. Proc Natl Acad Sci U S A.

[242] Ishikawa T, Kageyama S, Miyahara Y, Okayama T, Kokura S, Wang L (2021). Safety and antibody immune response of CHP-NY-ESO-1 vaccine combined with poly-ICLC in advanced or recurrent esophageal cancer patients. Cancer Immunol Immunother.

[243] Pavlick A, Blazquez AB, Meseck M, Lattanzi M, Ott PA, Marron TU (2020). Combined Vaccination with NY-ESO-1 Protein, Poly-ICLC, and Montanide Improves Humoral and Cellular Immune Responses in Patients with High-Risk Melanoma. Cancer Immunol Res.

[244] Kageyama S, Wada H, Muro K, Niwa Y, Ueda S, Miyata H (2013). Dose-dependent effects of NY-ESO-1 protein vaccine complexed with cholesteryl pullulan (CHP-NY-ESO-1) on immune responses and survival benefits of esophageal cancer patients. J Transl Med.

[245] Kitano S, Kageyama S, Nagata Y, Miyahara Y, Hiasa A, Naota H (2006). HER2-specific T-cell immune responses in patients vaccinated with truncated HER2 protein complexed with nanogels of cholesteryl pullulan. Clin Cancer Res.

[246] Schmid P, Adams S, Rugo HS, Schneeweiss A, Barrios CH, Iwata H, IMpassion130 Trial Investigators (2018). Atezolizumab and Nab-Paclitaxel in Advanced Triple-Negative Breast Cancer. N Engl J Med.

[247] Smith TT, Stephan SB, Moffett HF, McKnight LE, Ji W, Reiman D (2017). In situ programming of leukaemia-specific T cells using synthetic DNA nanocarriers. Nat Nanotechnol.

[248] Tang L, Zheng Y, Melo MB, Mabardi L, Castaño AP, Xie YQ (2018). Enhancing T cell therapy through TCR-signaling-responsive nanoparticle drug delivery. Nat Biotechnol.

